# Deciphering the Toxicity of Metal Tungstates and Molybdates: Effects on L929 Cell Metabolic Activity, Oxidative Stress, and Genotoxicity

**DOI:** 10.1002/jat.4836

**Published:** 2025-06-22

**Authors:** Marcelo Assis, Amanda de Souza, Karolyne dos Santos Jorge Sousa, Diana Gabriela Nina Nina, Mirian Bonfacio, Renata Neves Granito, Ana Claudia Muniz Rennó

**Affiliations:** ^1^ Department of Biosciences Federal University of São Paulo (UNIFESP) Santos SP Brazil

**Keywords:** genotoxicity, metabolic activity, metal molybdates, metal tungstates, oxidative stress

## Abstract

The increasing development and application of metal‐based materials in biomedical and environmental fields raise important concerns regarding their potential cytotoxic and genotoxic effects. Metal tungstates (M_x_WO_4_) and molybdates (M_x_MoO_4_) offer promising functional properties in health and environmental solutions but require safety validation before practical use. This study aimed to synthesize a series of these compounds based on Ag, Ca, Sr, and Zn and evaluate their behavior in both solid state and solution, focusing on their biological interactions with L929 fibroblast cells. Cell metabolic activity was assessed over 1, 3, and 7 days, revealing that Ag‐based materials were toxic even at low concentrations (7.8 μg/mL), while Ca‐, Sr‐, and Zn‐based compounds enhanced metabolic activity at lower doses. At concentrations above 62.5 μg/mL, Zn‐based materials showed toxicity, accompanied by morphological cell alterations. ROS production emerged as the primary mechanism of toxicity, especially for Ag‐based samples. Intracellular oxidative stress analysis confirmed elevated ROS and RNS levels over time. Apoptotic and necrotic pathways were identified only in α‐Ag_2_WO_4_ at the lowest dose. The micronucleus assay showed genotoxic responses in Ag‐based compounds comparable to positive controls, while other materials showed no significant genotoxicity. These findings indicate that Ca‐, Sr‐, and Zn‐based tungstates and molybdates may be safely applied in biological contexts, whereas Ag‐based materials, though effective, demand cautious use due to their long‐term genotoxic potential.

## Abstract 2

This study evaluates the cytotoxic and genotoxic profiles of Ag‐, Ca‐, Sr‐, and Zn‐based tungstates and molybdates. While Ag‐based compounds showed toxicity and genotoxicity at low concentrations, Ca‐, Sr‐, and Zn‐based materials enhanced fibroblast metabolic activity and showed minimal adverse effects. ROS generation was identified as the main toxicity mechanism. These results support the potential safe use of Ca‐, Sr‐, and Zn‐based materials in biomedical applications, highlighting the need for caution with Ag‐based systems due to genotoxic concerns.

**Abbreviations**
AO: acridine orange
DCFH‐DA: 2′,7′‐dichlorodihydrofluorescein diacetate
DMSO: dimethyl sulfoxide
EB: ethidium bromide
MMS: methyl methanesulfonate
MTT: 3‐(4,5‐dimethylthiazol‐2‐yl)‐2,5‐diphenyltetrazolium bromide
M_x_MoO_4_
: molybdates
M_x_WO_4_
: metal tungstates
NO_2_^−^
: nitrite ion
NOS: nitric oxide synthase
ONOO^−^
: peroxynitrite
⦁O_2_^−^
: superoxide radical
RNS: reactive nitrogen species
ROS: reactive oxygen species
SEM: scanning electron microscopy
XRD: X‐ray diffraction

## 1 Introduction

The advancement of materials plays a crucial role in driving innovation across various technological fields, from energy storage and electronics to healthcare and environmental remediation. As new materials are developed to meet the growing demands for efficiency, sustainability, and functionality, it becomes equally important to assess their safety and potential impacts (Cassee et al. 2024). Studying the toxicity of these materials is essential, particularly when they are intended for applications involving human health problems. Understanding the toxicological profiles of materials helps ensure that they do not pose risks to health, facilitating their safe and responsible integration into various technological sectors (Tralau et al. 2015). Additionally, toxicity studies provide valuable insights for designing more biocompatible and environmentally friendly materials, promoting sustainable technological progress (Buchman et al. 2019)

Within the field of advanced materials, the semiconductor market has been growing exponentially due to its multifunctionality (Yeboah et al. 2024). These materials are widely used in various applications, ranging from the development of new catalysts for energy generation to their use in biomaterials (Hochbaum and Yang 2010; Song et al. 2024; Tyczkowski and Kierzkowska‐Pawlak 2024). Their versatility makes them essential for a range of emerging technologies, such as renewable energy (Shah et al. 2023), electronic devices (Wang et al. 2021), and environmental purification systems (Xue et al. 2020). In this context, two types of semiconductors that have been gaining prominence are metal tungstates (M_x_WO_4_, M = metal) and molybdates (M_x_MoO_4_, M = metal) (Assis, Castro, et al. 2023; Costa et al. 2023; Gurusamy et al. 2024; Smith Pellizzeri et al. 2020). These materials possess unique properties that make them promising for applications like photocatalysis, sensors, and electronic devices, solidifying their role in advancing sustainable and innovative technologies.

Within the realm of transition metal tungstates, notable examples include Ag_2_WO_4_, CaWO_4_, SrWO_4_, and ZnWO_4_. Ag_2_WO_4_ stands out as one of the most versatile semiconductors among them, with applications ranging from sensors and catalysts to antimicrobial and antitumor agents (Assis et al. 2019; da Silva et al. 2016; Gouveia, Assis, et al. 2022; Onue et al. 2024; Patrocinio et al. 2023). It exists in three polymorphic forms, with the orthorhombic α‐Ag_2_WO_4_ being the most stable at room temperature and pressure (Alvarez‐Roca et al. 2021). CaWO_4_ and SrWO_4_, both possessing a tetragonal structure, are widely used as luminescent probes, pigments, and antimicrobial materials (Baby et al. 2023; Gouveia, Roca, et al. 2022). ZnWO_4_, with a monoclinic structure, is a key material for developing new photocatalysts and has demonstrated antiangiogenic properties (Pereira et al. 2018; Santos et al. 2018). Similarly, in the family of transition metal molybdates, Ag_2_MoO_4_, CaMoO_4_, SrMoO_4_, and ZnMoO_4_ are prominent. Ag_2_MoO_4_, which exists in two polymorphs, with the cubic (β) form being the most stable, is widely used for the development of photocatalysts, antimicrobial agents, and supercapacitors (De Foggi et al. 2020; Macchi et al. 2024; Teodoro et al. 2022). CaMoO_4_ and SrMoO_4_ also have a tetragonal structure, making them ideal hosts for rare earth elements in luminescent applications and photocatalysis (Bi et al. 2009; Gao et al. 2011; X. Li et al. 2011; Longo et al. 2011). ZnMoO_4_, like its tungstate counterpart, exists in two polymorphic forms, with the lower‐symmetry triclinic structure (β) being the most stable (Cavalcante et al. 2013). This material is similarly employed in photocatalysis and energy storage applications, such as battery development (Nunna et al. 2024). These materials are critical across numerous technological fields, from energy generation and storage to healthcare and environmental solutions. However, it is essential to conduct a systematic evaluation of their cytotoxic effects to ensure their safety and suitability for widespread use. By understanding their potential biological impacts, we can better harness their advanced properties while minimizing risks to human health and the environment.

In contact with biological systems, the impact of these semiconductors can manifest in various ways (Braga et al. 2024). The first mechanism involves physical interaction, where the surface charge of the semiconductor plays a crucial role in its interaction with negatively charged cell membranes (Fragelli et al. 2024). This surface charge can either promote or hinder cellular uptake processes. Secondly, the ionic release of corresponding metal ions from these semiconductors can have harmful or benign effects, depending on the ions involved. Ag^+^ is particularly known for its strong antimicrobial activity, making it effective in preventing infections in medical devices and implants (Sukhorukova et al. 2017). Meanwhile, Ca^2+^ and Sr^2+^ ions are generally well tolerated in controlled amounts, with Ca^2+^ being essential for bone mineralization and promoting osteogenesis, thereby enhancing the integration of biomaterials with bone tissue (Z. Li et al. 2021). In contrast, Sr^2+^ has shown potential in stimulating bone formation and inhibiting bone resorption, contributing positively to overall bone health (F. Chen, Tian, et al. 2022). Zn^2+^ is crucial in various biological processes, including wound healing and antioxidant defense, but can become toxic at elevated concentrations (Y. Chen, Cai, et al. 2022). Conversely, Mo^6+^ and W^6+^ ions typically pose fewer biological risks, with Mo^6+^ having the ability to acidify the medium (Assis et al. 2024; Fragelli et al. 2024). Lastly, due to their nature as semiconductors, these materials can exhibit defects in their structures that lead to the generation of reactive oxygen species (ROS) in contact with water and/or oxygen molecules, even in the absence of light (Grasser et al. 2025; Libero et al. 2023). If the production of ROS is significant, it can trigger oxidative stress and generate harmful radicals, potentially causing severe damage to cells and tissues (de Oliveira et al. 2023). However, in controlled concentrations, ROS can play a beneficial role by stimulating angiogenic processes and enhancing cellular regeneration (Yao et al. 2019). Thus, while ROS can be advantageous, excessive amounts can lead to toxicity, highlighting the need for careful evaluation in the context of biomaterial applications.

These multifunctional materials, already widely explored in sectors such as energy and environmental technologies, exhibit chemical and structural characteristics, such as ion release, surface reactivity, and semiconductor behavior, that are also highly relevant for biomedical applications. Their established functional performance in non‐biological systems raises the possibility of repurposing them for use in biomaterials, particularly in tissue engineering, regenerative medicine, and antimicrobial therapies. However, transferring these materials into biomedical contexts requires more than just performance‐based justification; it demands a rigorous assessment of their biological interactions. While some of their properties, like controlled ion release and ROS generation, may be beneficial in promoting healing or inhibiting microbial growth, these same features can also induce cellular stress, toxicity, or genotoxicity if not finely tuned. Therefore, understanding their safety profile is essential to unlock their full potential in biomedical applications.

In this work, it was hypothesized that metal‐based M_x_WO_4_ and M_x_MoO_4_ (M = Ag, Ca, Sr, and Zn) possess distinct biological responses that depend on their composition and concentration and that a thorough evaluation of their metabolic activity and genotoxic profiles is essential to determine their potential suitability for biomedical applications. By investigating these properties in detail, this study aims to identify which compositions could be safely explored in the design of future biomaterials. All materials were synthesized using microwave‐assisted hydrothermal techniques in an aqueous medium. The materials were characterized by X‐ray diffraction (XRD) and scanning electron microscopy (SEM), while their behavior in solution was analyzed through zeta potential, dynamic light scattering (DLS), and ionic release measured by ICP‐MS. MTT assays were performed at 1, 3, and 7 days to evaluate both acute and chronic exposure, using direct and indirect methods to effectively separate the toxic contributions of these materials. Optical microscopy was used to assess the integrity of cell morphology. In addition, the production of ROS and reactive nitrogen species (RNS) was measured in L929 cells using 2′,7′‐dichlorodihydrofluorescein diacetate (DCFH‐DA) and the Griess reaction, respectively. Genotoxicity analysis was conducted using the micronucleus test in CHO‐K1 cells, while cell death type and nuclei fragmentation were assessed through the acridine orange (AO) and ethidium bromide (EB) using L929 cells. Based on these results, the study systematically sought to explain the cytotoxic effects caused by this class of materials, aiming to adapt insights on environmental safety regarding their use.

## 2 Materials and Methods

### 2.1 Synthesis

The reagents used for the synthesis were AgNO_3_ (Cennabras, 99.8%), Ca (NO_3_)_2_·4H_2_O (Sigma‐Aldrich, 99%), Sr (NO_3_)_2_ (Sigma‐Aldrich, > 99.0%), Zn (NO_3_)_2_·6H_2_O (Sigma‐Aldrich, 98%), Na_2_WO_4_·2H_2_O (Sigma‐Aldrich, > 99%), and Na_2_MoO_4_ (Sigma‐Aldrich, > 98%). All materials were synthesized via a coprecipitation method in an aqueous medium, followed by microwave‐assisted hydrothermal treatment. A solution was prepared by dissolving 1 × 10^−3^ mol of the W/Mo reagent in 50 mL of distilled water. In a separate beaker, a stoichiometric quantity of the corresponding nitrate salt (1 × 10^−3^ mol for Ca, Sr, and Zn; 2 × 10^−3^ mol for Ag) was dissolved in another 50 mL of distilled water. Both solutions were heated to 70°C, after which the nitrate solution was rapidly introduced into the W/Mo solution under continuous stirring. An immediate precipitation occurred, and the mixture was kept under stirring for 20 min. The suspension was then transferred into a Teflon‐lined autoclave, ensuring no magnetic stirring, and subjected to microwave treatment (2.45 GHz, maximum power of 800 W) at 160°C for 32 min. The obtained precipitate was thoroughly washed with distilled water 10 times to eliminate residual counterions and subsequently dried in an oven at 60°C for 12 h.

### 2.2 Characterizations

XRD analysis was performed on powdered samples using a Bruker D4‐Endeavor diffractometer with Cu Kα radiation (*λ* = 1.5406 Å). The data were collected over a 2θ range of 10° to 70°, with a step size of 0.02°, allowing for precise identification of crystalline phases. Morphological characterization was carried out by SEM using a LEO 440i Leica‐Zeiss microscope operating at 10 kV. For SEM analysis, the samples were initially dispersed in distilled water with the aid of an ultrasonic bath to promote particle separation. A drop of the resulting suspension was then deposited onto a silicon substrate and left to dry at room temperature. Zeta potential and DLS analyses were performed in triplicate for each sample. The materials were dispersed in aqueous solution, sonicated to ensure homogeneity, and analyzed using a Zetasizer NanoZS (Malvern, UK). Zeta potential measurements were conducted at different pH values, adjusted with NaOH (24%, Synth) and HNO_3_ (37%, Synth). DLS measurements were carried out in both distilled water and Dulbecco's Modified Eagle's Medium (DMEM, VitroCell) to evaluate the hydrodynamic particle size under conditions relevant to biological systems. All analyses were performed for each of the synthesized compounds to ensure a comprehensive comparison of their physicochemical properties. The metal quantification was performed using ICP‐OES iCAP 7000 (Thermo Fischer Scientific).

### 2.3 Cell Culture

This study utilized the L929 murine fibroblast cell line, and all tests were conducted in accordance with the OECD Guidance Document on Good In Vitro Method Practices (OECD 2018). The cells were maintained in culture flasks containing DMEM medium (VitroCell) supplemented with 10% heat‐inactivated fetal bovine serum (FBS) (VitroCell). Incubation was carried out at 37°C in a humidified atmosphere with 5% CO_2_ until the cultures reached approximately 80% confluence, with passaging performed as needed. Cells were exposed to the materials under both direct and indirect contact conditions at concentrations of 1.9, 3.9, 7.8, 15.6, 31.2, 62.5, 125, 250, 500, and 1000 μg/mL for 1, 3, and 7 days. In the direct exposure test, the material was dispersed directly into the medium and added to the cell culture. For the indirect exposure assays, extracts of the samples were prepared by dispersing the powdered materials in standard culture medium at a concentration of 1000 μg/mL. The suspensions were incubated for 24 h in a humidified atmosphere at 37°C with 5% CO_2_ to allow the release of soluble components. Following incubation, the extracts were filtered through a 0.22‐μm membrane filter (Kasvi, Curitiba, Brazil) to remove any residual particles and were subsequently used in the biological tests without further dilution. Images were acquired using a LOD‐3000 Zeiss microscope equipped with a 12 MP camera.

Various biomarkers can be used to assess the toxicological effects of nanomaterials, as different cellular components may respond in distinct ways to external stimuli. Oxidative and nitrosative stress, driven by the intracellular accumulation of reactive oxygen (ROS) and nitrogen species (RNS), plays a fundamental role in how cells respond to nanomaterials (Aranda‐Rivera et al. 2022; Makhdoumi et al. 2020). The levels of ROS/RNS generated are crucial, as mild and transient increases can activate cellular defense mechanisms and lead to adaptation, while excessive or sustained production may cause progressive damage to proteins, lipids, and DNA (Pickering et al. 2013). To capture these different layers of response, it is necessary to combine complementary assays that assess distinct but interconnected cellular pathways. The MTT (3‐(4,5‐dimethylthiazol‐2‐yl)‐2,5‐diphenyltetrazolium bromide) assay reflects mitochondrial metabolic activity and serves as a general indicator of cell viability (Surin et al. 2017). The acridine orange/ethidium bromide (AO/EB) staining assay provides a morphological evaluation of plasma membrane integrity and enables the distinction between viable, apoptotic, and necrotic cells (Atale et al. 2014). Understanding the predominant mode of cell death is essential, as it offers insight into the severity and mechanism of cellular damage. While apoptosis often reflects a regulated and potentially reversible response to stress, necrosis may indicate acute and irreversible injury (FADEEL and ORRENIUS 2005; Ude et al. 2022). Importantly, the micronucleus assay was included to detect chromosomal damage, offering valuable information on potential genotoxic effects (Fenech 2008). Even in cases where metabolic activity and membrane integrity appear unaffected, DNA damage may still occur, making this endpoint critical for a complete evaluation of cellular responses. This combined strategy enables a more robust and nuanced interpretation of how cells interact with the tested materials over time.

### 2.4 Metabolic Activity

The MTT colorimetric assay (Sigma‐Aldrich) was conducted to evaluate mitochondrial function integrity, based on the reduction of MTT to formazan crystals by mitochondrial enzymes. This test was performed based on the guidelines established in ISO 10993‐5:2009 for cytotoxicity assessment (Tests for in vitro cytotoxicity). L929 cells were seeded at a density of 1 × 10^4^ cells per well in a flat‐bottomed 96‐well microplate pre‐treated for cell culture and covered with a lid. After 24 h of adhesion, the materials were introduced into the wells. Following exposure periods of 1, 3, and 7 days, the wells were washed with phosphate‐buffered saline (PBS, VitroCell), and 50 μL of MTT solution (0.5 mg/mL in PBS) was added to each well. The reaction was allowed to proceed for 4 h at 37°C in a 5% CO_2_ atmosphere. Blank controls containing only MTT solution were included in the assay. After incubation, the reagent solution was carefully removed, and 100 μL of isopropanol was added to each well to dissolve the formazan crystals. Absorbance was then measured at 570 nm using a BioTek Instruments Microplate Spectrophotometer. All tests were performed in triplicate on three independent occasions.

### 2.5 Intracellular ROS Probe

ROS production was assessed using the fluorescent probe DCFH‐DA (Sigma‐Aldrich). L929 cells were seeded in black 96‐well plates under the same conditions described previously and exposed to the material at the concentration that demonstrated the best performance in the prior assay (1.9–7.8 μg/mL). After 24 h of adhesion, the materials were introduced into the wells. Following exposure, the wells were washed twice with PBS (VitroCell), and 100 μL of a 100‐μM DCFH‐DA solution prepared in PBS was added. The reaction was allowed to proceed for 30 min in a humidified chamber at 37°C with 5% CO_2_, protected from light. The reagent solution was then removed, and the wells were washed with PBS before adding another 100 μL of PBS per well. Fluorescence intensity was recorded at an excitation/emission wavelength of 485/530 nm using a BioTek Instruments Microplate Fluorometer. The experiments were performed in triplicate on three independent occasions.

### 2.6 Intracellular RNS Probe

RNS production was evaluated using the Griess reaction, which quantifies nitrite ion (NO_2_^−^) formation based on its reaction with sulfanilamide in an acidic medium. L929 cells were seeded in a flat‐bottomed 96‐well microplate under the same conditions previously described and exposed to the material at the concentration that yielded the best results in the preceding assay (1.9–7.8 μg/mL). After 24 h of adhesion, the materials were introduced into the wells. Following a 1, 3, and 7 days of exposure period, 50 μL of the supernatant was collected and transferred to a new plate, where 50 μL of the Griess reagent was added. This reagent consisted of a 1:1 mixture of Solution A (1% sulfanilamide in 5% phosphoric acid) and Solution B (0.1% *N*‐(1‐naphthyl)ethylenediamine dihydrochloride). The reaction was allowed to proceed for 15 min at room temperature, shielded from light. Absorbance was measured at 540 nm using a BioTek Instruments Microplate Spectrophotometer. The nitrite concentration in the supernatant was determined using a standard curve with known nitrite concentrations (nM), following the protocol outlined in the Griess reagent (modified) kit (Sigma‐Aldrich, G4410). All experiments were conducted in triplicate on three independent occasions.

### 2.7 Apoptosis and Necrosis Assays

L929 cells were seeded in black 96‐well plates under the same conditions previously described and exposed to the material at the concentration that yielded the best results in the prior assay. After 24 h of exposure at 3.9 μg/mL of the materials, the cells were washed with PBS and stained with a mixture of AO/EB (1 mg/mL:1 mg/mL) for 15 min in the dark. Following staining, the cells were thoroughly washed to remove excess dye. The evaluation of apoptotic and necrotic cells, along with morphological analysis, was carried out using an ImageXpress Micro (Molecular Devices) equipped with an excitation filter of 515–560 nm and a barrier filter of 590 nm. All experiments were conducted in triplicate.

### 2.8 Micronucleus Assays

The micronucleus assay was conducted in three independent experimental replicates using the CHO‐K1 cell line, following the guidelines of OECD 487 (OECD 2023). Initially, 0.5 × 10^6^ cells were seeded into six‐well culture plates and incubated for 24 h to allow cell adhesion. Following this period, CHO‐K1 cells were exposed to the materials at a concentration of 3.9 μg/mL for 4 h. After exposure, the culture medium was replaced with fresh medium containing Cytochalasin B (3 μg/mL, Sigma‐Aldrich), and the cells were incubated for an additional 24 h. After incubation, the cells were washed twice with PBS, trypsinized, and centrifuged in 15 mL tubes at 1500 rpm for 5 min. The pellet was resuspended in a cold hypotonic solution (1% sodium citrate at 4°C, Sigma‐Aldrich) and 25% formaldehyde for 4 min, followed by centrifugation under the same conditions. Next, the cells were resuspended, fixed, and centrifuged twice for 5 min in a methanol/acetic acid solution (3:1 v/v, Sigma‐Aldrich). The resulting cell suspension was transferred onto pre‐cleaned slides, which were then stained using a rapid panoptic staining kit (New Prov, Paraná, Brazil). Once dried, the slides were analyzed under a light microscope (Nikon) at 630× magnification, with a total of 1000 binucleated cells counted per sample. The culture medium served as the negative control, 0.5% dimethyl sulfoxide (DMSO) was used as the solvent control, and 40 μM methyl methanesulfonate (MMS) was used as the positive control. All tests were performed in triplicate on two separate occasions.

### 2.9 Statistical Analysis

All statistical analyses were performed using the software GraphPad Prism version 9.0. Initially, potential outliers were identified using Grubbs' test. To determine the appropriate statistical approach, the normality of the data distribution was evaluated using the Shapiro–Wilk test. In cases where the data followed a normal distribution, one‐way analysis of variance (ANOVA) was applied, followed by Tukey's post hoc test for multiple comparisons, with results expressed as mean ± standard deviation. For data that did not meet the assumptions of normality, the Kruskal–Wallis test was used, followed by Dunn's post hoc test, with results expressed as median and interquartile range. A significance level of *p* ≤ 0.05 and *p* ≤ 0.01 was adopted for all analyses.

## 3 Results and Discussion

### 3.1 Characterizations

**FIGURE 1:**
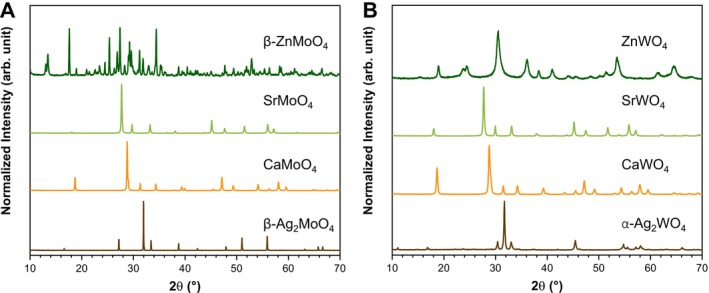
XRD patterns of metal (A) molybdates and (B) tungstates.

The success of the synthesis was first evaluated through long‐range structural analysis using XRD (Figure 1). Ag_2_MoO_4_ was identified as having an inverted cubic spinel structure with space group *Fd‐3m*, corresponding to the thermodynamically stable β‐polymorph (β‐Ag_2_MoO_4_) (Macchi et al. 2024). Both CaMoO_4_ and SrMoO_4_ were characterized by their tetragonal structure with space group *I41/a* (Mikhailik et al. 2007; Sczancoski et al. 2008), while ZnMoO_4_ exhibited a low symmetric triclinic hydrated structure with space group *P1*, corresponding to its most stable β‐polymorph (β‐ZnMoO_4_) (Zhang et al. 2010). For the tungstates, Ag_2_WO_4_ was identified with an orthorhombic structure and space group *Pn2n*, consistent with its stable α‐polymorph (α‐Ag_2_WO_4_) (Assis, Gouveia, et al. 2023). As seen with the molybdates, CaWO_4_ and SrWO_4_ also possess a tetragonal structure with space group *I41/a* (Gouveia, Assis, et al. 2022; Sczancoski et al. 2009), while ZnWO_4_ was characterized by a monoclinic structure and space group *P2/c* (Gondim et al. 2021). No secondary phase formation was observed in any of the synthesized materials, highlighting the success of the microwave‐assisted hydrothermal synthesis. All materials exhibited sharp and well‐defined diffraction peaks, indicating high crystallinity, except for ZnWO_4_, which displayed peak broadening due to its nanometric morphology.

**FIGURE 2:**
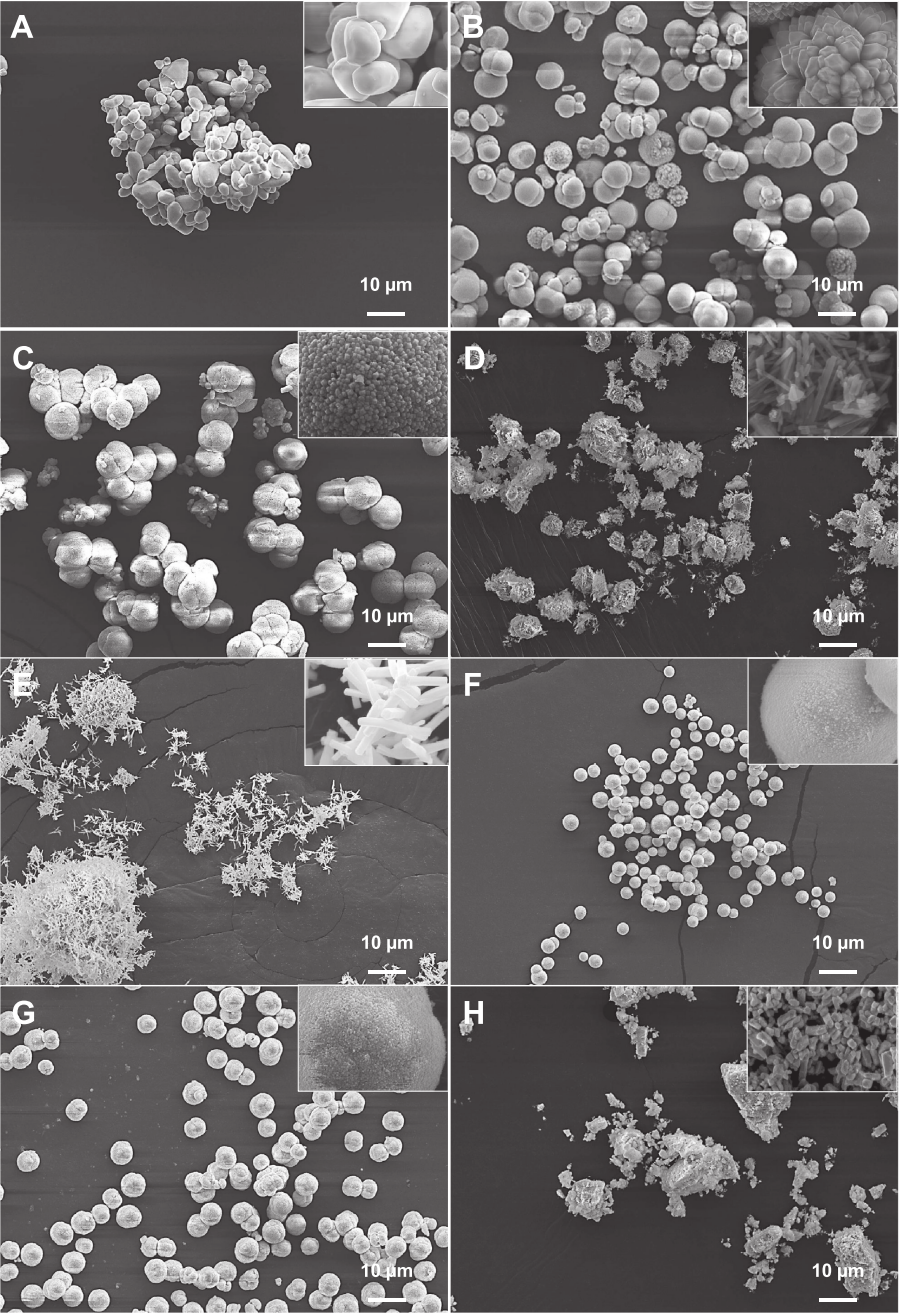
SEM images of the samples (A) β‐Ag_2_MoO_4_, (B) CaMoO_4_, (C) SrMoO_4_, (D) β‐ZnMoO_4_, (E) α‐Ag_2_WO_4_, (F) CaWO_4_, (G) SrWO_4_, and (H) ZnWO_4_.

The morphology and size of the synthesized particles, analyzed by SEM (Figure 2), play a critical role in determining how these particles will interact with cells, particularly regarding their potential for cellular uptake (Mailänder and Landfester 2009). The size of the particles is a key factor that influences whether cells can internalize them, as smaller particles are more likely to be taken up by cells, while larger particles may remain on the cell surface. According to previous studies, L929 cells typically range in size from 5 to 15 μm (Higuchi and Tsukamoto 2004). Particles that are smaller than the cell size, especially those in the submicron or nanometric range, are more easily internalized via endocytosis or phagocytosis (Baranov et al. 2021), making them potentially more effective for certain applications like drug delivery or intracellular sensing. Conversely, larger particles may interact with the cell membrane but are less likely to be internalized (Ma et al. 2013), which can be advantageous for applications where surface interactions are critical, such as in biosensing or external scaffolding. Therefore, the particle size not only affects the degree of cellular uptake but also the intended biological function of the materials.

The β‐Ag_2_MoO_4_ exhibited deformed spheroidal morphologies with an average diameter of 4.099 ± 1.089 μm. CaMoO_4_ mostly exhibited a spheroidal morphology, with an average diameter of 7.395 ± 0.885 μm. However, in some cases, dumbbell shapes were observed, representing early stages of the self‐assembly of CaMoO_4_ spheroids (Gouveia, Roca, et al. 2022). SrMoO_4_ also displayed a spheroidal morphology, possessing the largest particle size among the samples, with an average diameter of 8.803 ± 0.547 μm. β‐ZnMoO_4_ presented a rod‐like morphology, with an average length of 0.468 ± 0.107 μm and a width of 0.124 ± 0.029 μm. For α‐Ag_2_WO_4_, hexagonal rods were obtained, with an average length of 1.313 ± 0.319 μm and a width of 0.251 ± 0.035 μm. CaWO_4_ and SrWO_4_ displayed spherical morphologies with average diameters of 3.709 ± 0.552 and 5.719 ± 0.647 μm, respectively. ZnWO_4_ was the only material synthesized by the microwave‐assisted hydrothermal process that exhibited nanometric morphology. Its rod‐shaped structures had an average length of 40.2 ± 4.7 nm and an average width of 15.1 ± 6.2 nm. In this context, the analysis of particle size reveals that the only particles likely to undergo cellular uptake are β‐ZnMoO_4_, α‐Ag_2_WO_4_, and ZnWO_4_. However, Fragelli et al. found that hexagonal rods of α‐Ag_2_WO_4_ were not internalized in 3T3 murine cells, as demonstrated through flow cytometry tests, where the particles were labeled with rhodamine B (Fragelli et al. 2024). This suggests that, despite their size, α‐Ag_2_WO_4_ rods may not be efficiently taken up by cells, in particular due to its negative surface charge. Consequently, the only viable candidates for cellular internalization are the Zn‐based particles, which exhibit the smallest dimensions within their respective groups.

Once the particle sizes were analyzed in their solid form, their behavior in solution was assessed using DLS. This analysis is crucial because, in liquid environments that resemble biological conditions, the physical and chemical properties of particles can change, affecting their interaction with cells (Mu et al. 2014). In solution, particles may aggregate or disperse differently depending on factors like pH and surface charge, which can influence cellular interaction. Additionally, surface chemistry may be altered by the adsorption of biomolecules, impacting how particles are recognized by cells (Verma and Stellacci 2010). Figure S1 shows the hydrodynamic radius of the particles in water and in DMEM culture medium. For the larger samples, not much difference is observed between their sizes measured by SEM and their hydrodynamic size, except for β‐Ag_2_MoO_4_, which nearly doubles in size, indicating that this material likely aggregates in aqueous solutions. Significant differences from their SEM sizes are observed for the smaller samples, specifically β‐ZnMoO_4_, α‐Ag_2_WO_4_, and ZnWO_4_, which have hydrodynamic sizes of 1.198 ± 0.802, 0.746 ± 0.339, and 0.248 ± 0.106 μm in water, and 1.528 ± 1.389, 1.689 ± 0.895, and 0.597 ± 0.287 μm in DMEM, respectively. These results show that in solutions, these materials undergo significant aggregation and do not behave as individual particles.

As previously mentioned, the surface charge of particles can significantly influence not only their aggregation behavior but also their interaction with cells. The surface charge affects how particles interact with the cell membrane, which typically carries a negative charge (Fröhlich 2012). Positively charged particles may exhibit stronger interactions with the negatively charged cell membrane, while negatively charged particles may experience repulsion, thus reducing cellular internalization. To analyze the surface charge of the particles, zeta potential analyses were conducted and are shown in Figure S2. All materials exhibit a similar surface charge profile, becoming more negative at basic pH and more positive at acidic pH. At pH 7 (close to the pH 7.4 of DMEM), the samples β‐Ag_2_MoO_4_, CaMoO_4_, SrMoO_4_, and β‐ZnMoO_4_ show zeta potentials of −37.8, −15.4, −34.1, and −18.9 mV, respectively. For the tungstates, α‐Ag_2_WO_4_, CaWO_4_, SrWO_4_, and ZnWO_4_, the zeta potentials are −29.3, −39.7, −22.8, and −15.6 mV, respectively. Therefore, strong electrostatic interactions between the material particles and cell membranes are unlikely, as both carry negative surface potentials.

### 3.2 Cellular Assays

#### 3.2.1 Metabolic Activity

**FIGURE 3:**
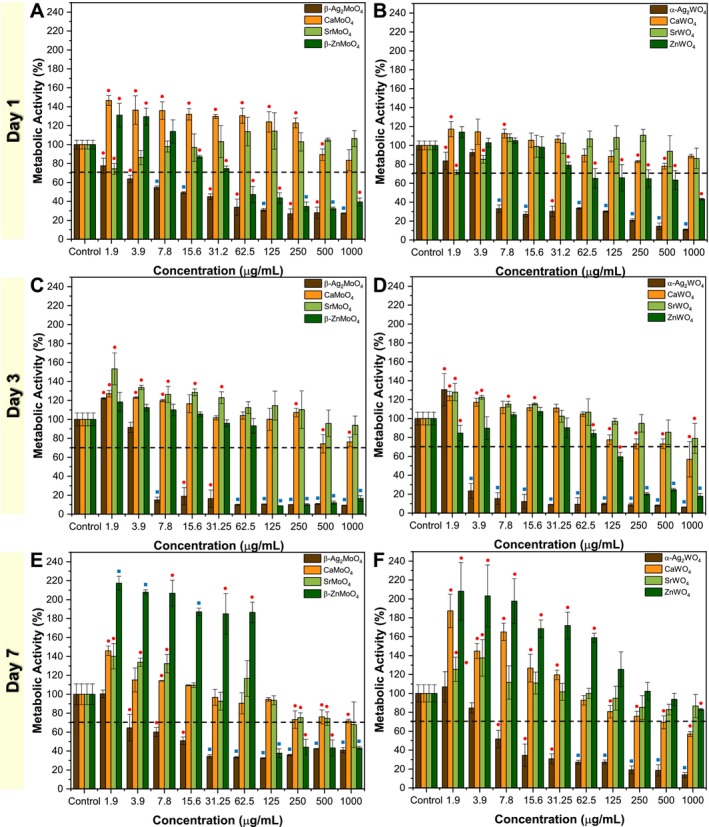
Cytotoxic MTT assay via direct contact using L929 cells: evaluation of metal molybdates at (A) 1, (C) 3, and (E) 7 days, and metal tungstates at (B) 1, (D) 3, and (F) 7 days. (●/■) versus Control: ● *p* ≤ 0.05; ■ *p* ≤ 0.01.

The materials analyzed here are semiconductors, which, as highlighted in the introduction, can interact electrostatically with cells, as well as produce ROS and release ions. Through previous analyses, it is observed that the electrostatic interaction between the materials and cells is not favored. The MTT assay is based on the reduction of the tetrazolium salt MTT, resulting in a blue‐violet product proportional to the number of viable cells (Brassolatti et al. 2022). Although this method specifically measures cellular metabolic activity, a decrease in the results is commonly associated with higher cytotoxicity, while an increase may reflect improved cell viability or stimulation of cellular functions. In this study, we performed the assays at intervals of 1, 3, and 7 days using a serial dilution (1000–1.9 μg/mL) to evaluate both acute and chronic effects of exposure to the materials. This approach allows us to capture the dynamics of cellular response over time, revealing potential changes in cytotoxicity mechanisms. The metabolic activity assays were conducted directly with L929 fibroblasts by adding the particles directly to the cells, revealing different profiles for each material (Figure 3).

For β‐Ag_2_MoO_4_, metabolic activity during direct contact is strongly concentration dependent. On Day 1, only the concentration of 1.9 μg/mL is considered nontoxic, with cell viability above 70% according to ISO 10993‐5:2009 standards. All tested concentrations showed statistically significant differences compared with the control group. By Day 3, concentrations of 1.9 and 3.9 μg/mL remained nontoxic, while all higher concentrations resulted in cell viability dropping below 10%. Among these, only the 3.9 μg/mL concentration did not show a significant difference from the control. On Day 7, results mirrored those of Day 1, with 3.9 μg/mL again being the only concentration not significantly different from the control. For indirect contact tests (Figure S3), which assess the effects of ionic release from the material, concentrations above 31.2 μg/mL were found toxic on Day 1, with all concentrations showing significant differences from the control. However, on Day 3, no concentration was toxic, and only the 1.9 μg/mL concentration showed a slight increase in metabolic activity compared with the control. By Day 7, cytotoxicity was observed only at the highest concentration of 1000 μg/mL, while 1.9 μg/mL was the only concentration not significantly different from the control. Direct contact exposure was associated with pronounced cellular stress, reflected in morphological changes from the typical spindle shape to a spherical morphology, indicative of severe stress (Figure S4). In contrast, such morphological alterations were absent during indirect contact, suggesting that ionic mechanisms exert less aggressive effects than those involving direct particle exposure (Figure S5).

For α‐Ag_2_WO_4_, results similar to those for β‐Ag_2_MoO_4_ were observed, indicating that the lattice formers (Mo^6+^ and W^6+^) do not significantly influence the metabolic activity of these materials. In direct contact assays, on Day 1, only the concentrations of 1.9 and 3.9 μg/mL were nontoxic, with only 3.9 μg/mL showing no significant differences compared with the control. On Day 3, 1.9 μg/mL was the sole nontoxic concentration, with all other results significantly differing from the control. By Day 7, a pattern similar to Day 1 emerged, with 1.9 and 3.9 μg/mL being nontoxic and not significantly different from the control. For indirect contact assays, on Day 1, concentrations above 31.2 μg/mL reached toxic levels, with all results significantly differing from the control. By Day 3, the metabolic activity threshold increased to 125 μg/mL, with concentrations above this value showing significant differences from the control. On Day 7, the results mirrored those of Day 1. Morphological changes in L929 cells were evident at concentrations exceeding the cytotoxic thresholds in both direct (Figure S6) and indirect (Figure S7) contact assays. Once again, direct contact exposure was substantially more aggressive than indirect contact, suggesting that the primary toxicity mechanisms involve ROS production by the materials coupled with physical interactions.

For CaMoO_4_, across all days of the direct contact assays, cell metabolic activity values consistently remained above 70% across all tested concentrations, showing a dose‐dependent response. However, on Day 1, concentrations below 500 μg/mL notably stimulated the metabolic activity of L929 cells, with the lowest concentrations resulting in metabolic activities exceeding 140%, which were significantly higher than the control. On Days 3 and 7, this metabolic stimulation was less pronounced, primarily observed at concentrations between 1.9 and 7.8 μg/mL. Particularly on Day 1, morphological alterations in the cells were evident, suggesting that despite the metabolic stimulation, the cells were still under stress (Figure S8). In indirect contact assays, no significant differences from the control were observed on Day 1 at any concentration. By Day 3, a slight stimulation of metabolic activity was noted at concentrations between 1.9 and 7.8 μg/mL, as well as at the highest concentration of 1000 μg/mL. A similar trend was observed on Day 7. Morphological changes were also noted on Day 1 in indirect contact tests, but the cells recovered their typical morphology by Days 3 and 7 (Figure S9). These findings highlight a complex interplay between metabolic activity stimulation and cellular stress, with the impact varying over time and between direct and indirect exposure scenarios.

For CaWO_4_, dose‐dependent behavior under direct contact was observed, with similar patterns on Days 1 and 3. On Day 1, no significant reductions in metabolic activity below 70% were detected. However, on Day 3, the concentration of 1000 μg/mL exhibited a metabolic activity below this threshold, with significant differences compared with the control observed at concentrations of 1.9, 3.9 μg/mL, and between 125 and 1000 μg/mL. By Day 7, a pronounced stimulation of cellular metabolic activity was noted at concentrations below 31.25 μg/mL, reaching values exceeding 180% at 1.9 μg/mL. This stimulation was dose‐dependent, with only the 1000 μg/mL concentration resulting in metabolic activity below 70%. For indirect contact assays, results were generally consistent across time points. On Day 1, only the 1.9 and 3.9 μg/mL concentrations showed a significant increase in metabolic activity compared with the control. On Day 3, a slight increase was observed at 7.8 μg/mL, while a slight but significant decrease was noted at 100 μg/mL. On Day 7, significant increases in metabolic activity were recorded at 500 and 100 μg/mL concentrations. Like CaMoO_4_, morphological changes were observed under both direct and indirect contact conditions. Cells adopted a globular morphology on the first day of exposure, which recovered to their typical appearance in subsequent experimental time points (Figures S10 and S11). These findings suggest cellular stress in early stages and a tendency for recovery over time.

Both Sr‐based materials (SrMoO_4_ and SrWO_4_) exhibit similar behaviors over time. Under direct contact conditions, on Day 1, SrMoO_4_ shows a significant reduction in cell viability only at the concentration of 1.9 μg/mL, while SrWO_4_ exhibits this reduction at concentrations of 1.9 and 3.9 μg/mL. By Day 3, SrMoO_4_ demonstrates a significant increase in metabolic activity at concentrations below 31.25 μg/mL, whereas SrWO_4_ shows a similar increase below 15.6 μg/mL. At higher concentrations, no significant changes are observed compared with the control. On Day 7, SrMoO_4_ induces a rise in metabolic activity at concentrations below 7.8 μg/mL, with significant reductions in viability only at 250 and 500 μg/mL. For SrWO_4_, increased metabolic activity is observed at 1.9 and 3.9 μg/mL, with no significant differences at other concentrations. Regarding cellular morphology, SrMoO_4_ causes morphological changes only on Day 1 at concentrations above 3.9 μg/mL. In contrast, SrWO_4_ induces morphological alterations across all concentrations on Day 1, and at concentrations above 125 μg/mL on Days 3 and 7 (Figures S12 and S14). For indirect contact, SrMoO_4_ shows no significant differences in metabolic activity at any concentration on Days 1 and 3. However, on Day 7, a slight increase in metabolic activity is observed at concentrations above 250 μg/mL. Morphological changes appear at concentrations above 1.9 μg/mL, as seen with direct contact (Figure S13). In contrast, SrWO_4_ exhibits distinct behavior under indirect contact conditions, with a significant increase in metabolic activity between 15.6 and 500 μg/mL on Day 3, followed by a decrease at 1000 μg/mL. Morphological changes are observed at all concentrations on Day 1, and above 500 μg/mL on Days 3 and 7 (Figure S15).

Like Sr‐based materials, Zn‐based materials (β‐ZnMoO_4_ and ZnWO_4_) exhibit a similar behavior under direct contact. On Day 1, β‐ZnMoO_4_ demonstrates a dose‐dependent effect, with a significant increase in metabolic activity at concentrations of 1.9 and 3.9 μg/mL. However, this activity decreases significantly from 15.6 μg/mL onward, reaching toxic levels at concentrations of 62.5 μg/mL and higher. On Day 3, no significant differences from the control are observed up to 62.5 μg/mL, but concentrations above this threshold result in drastic toxicity. Interestingly, on Day 7, concentrations below 62.5 μg/mL show a marked increase in metabolic activity, exceeding 190%, whereas concentrations above this level remain toxic. Morphological changes are evident at all concentrations on Day 1. By Days 3 and 7, such changes are observed only at 62.5 μg/mL, with higher concentrations showing no intact cells (Figure S16). For indirect contact, no significant differences from the control are observed on Day 1 at any concentration, although cells appear globular rather than spindle‐shaped (Figure S17). By Day 3, a substantial increase in metabolic activity is seen at concentrations below 250 μg/mL, similar to the direct contact results on Day 7. However, concentrations at or above 250 μg/mL exhibit a sharp decline in viability, indicating toxicity. On Day 7, no significant differences from the control are observed at concentrations below 500 μg/mL, but higher concentrations show pronounced toxicity.

Regarding ZnWO_4_, on Day 1, no significant differences are observed compared with the control up to 15.6 μg/mL. Above this concentration, viability decreases significantly, with all concentrations above 62.5 μg/mL considered toxic. On Day 3, a slight but significant reduction in viability is noted at 1.9 μg/mL, and toxic effects emerge at concentrations of 125 μg/mL and higher. Similar to β‐ZnMoO_4_, Day 7 shows a substantial increase in cell activity, exceeding 150% at concentrations below 125 μg/mL. At higher concentrations, none are considered toxic. Globular cell morphology is observed at all concentrations on Day 1, at concentrations above 15.6 μg/mL on Day 3, and at concentrations starting from 250 μg/mL on Day 7 (Figure S18). For indirect contact, ZnWO_4_ shows no major differences from the control on Day 1, except for slight increases in metabolic activity at 1.9, 31.25, 500, and 1000 μg/mL. By Day 3, all tested concentrations significantly enhance cell metabolic activity, exceeding 150% across the board. On Day 7, a modest but significant increase is observed at concentrations starting from 125 μg/mL. Morphological changes in L929 cell spindle shape are observed across all concentrations on Day 1 during indirect contact experiments (Figure S19).

As previously mentioned, in direct contact scenarios, two primary mechanisms are considered: ROS production and ionic release. In contrast, in indirect contact situations, only ionic release is taken into account. Regarding the lattice‐forming ions (W^6+^ and Mo^6+^), few studies report adverse effects at low concentrations. However, their main impact lies in the acidification of the medium, which may lead to cytotoxic effects due to the formation of soluble WO_4_^2−^ and MoO_4_^2−^ (Assis et al. 2024). To address this, pH measurements were conducted during the experimental periods analyzed (Figure S20). The data indicate a slight pH decrease across most materials (6.8–7.3), with a more pronounced effect observed for Ag‐based samples. This moderate drop in pH is consistent with the increased Mo^6+^/W^6+^ release observed for these materials shown in Table 1 and in Table S1 and S2.

**TABLE 1:** Ionic concentrations (μg/mL) released into the medium after 24 h of exposure for the different materials, measured by ICP analysis at the three lowest tested concentrations.

Concentration (μg/mL)	β‐Ag_2_MoO_4_		CaMoO_4_		SrMoO_4_		β‐ZnMoO_4_	
	Ag (μg/mL)	Mo (μg/mL)	Ca (μg/mL)	Mo (μg/mL)	Sr (μg/mL)	Mo (μg/mL)	Zn (μg/mL)	Mo (μg/mL)
1.9	0.1423	0.1856	0.0212	0.0398	0.0090	0.0169	0.0249	0.0356
3.9	0.2322	0.4162	0.0522	0.0859	0.0127	0.0428	0.0366	0.0628
7.8	0.3982	0.7582	0.0925	0.1253	0.0418	0.0698	0.0534	0.0821

Concentration (μg/mL)	α‐Ag_2_WO_4_		CaWO_4_		SrWO_4_		ZnWO_4_	
	Ag (μg/mL)	W (μg/mL)	Ca (μg/mL)	W (μg/mL)	Sr (μg/mL)	W (μg/mL)	Zn (μg/mL)	W (μg/mL)
1.9	0.2644	0.3289	0.0987	0.1468	0.0198	0.0389	0.0312	0.0356
3.9	0.3180	0.7526	0.1426	0.2750	0.0265	0.0548	0.0425	0.0774
7.8	0.5437	1.0735	0.2012	0.3972	0.0399	0.0724	0.0617	0.0973

For lattice‐modifying ions (Ag^+^, Ca^2+^, Sr^2+^, and Zn^2+^), their effects differ substantially. At low doses, Ag^+^, a nonessential ion, can activate signaling pathways that enhance cell survival (Duan et al. 2018). However, such concentrations are typically very low (nM to low μM, depending on the cell type). Conversely, Ca^2+^, an essential ion, promotes critical cellular signaling processes necessary for cell survival and differentiation (Patergnani et al. 2020). It also regulates cell adhesion and extracellular matrix remodeling (Ermak and Davies 2002). However, high micromolar concentrations can trigger apoptosis in cells. Sr^2+^, which shares similarities with Ca^2+^, shows its most beneficial effects in bone tissues, where it enhances osteoblast proliferation and differentiation and modulates cellular signaling pathways (You et al. 2022). Yet, concentrations exceeding several dozen micromolar can induce cytotoxicity. Zn^2+^, on the other hand, stimulates tissue proliferation and cellular regeneration at concentrations generally below 50 μM (Y. Li and Maret 2009). Additionally, Zn^2+^ enhances antioxidant defenses by activating enzymes like superoxide dismutase (Craven et al. 2001).

In indirect contact experiments, materials that significantly alter the metabolic activity of L929 cells are primarily those based on Ag (α‐Ag_2_WO_4_ and β‐Ag_2_MoO_4_) and Zn (ZnWO_4_ and β‐ZnMoO_4_). On the first day of indirect exposure to β‐Ag_2_MoO_4_, the ionic release, particularly of Ag^+^, reaches critical levels that reduce cell viability. However, cells recover from this damage over time. In contrast, α‐Ag_2_WO_4_ consistently exhibits unrecoverable toxic levels at all experimental periods above 31.2 μg/mL, indicating that α‐Ag_2_WO_4_ releases more Ag^+^ than β‐Ag_2_MoO_4_. To better understand the contribution of ionic release to the observed biological effects, the leached ionic species were analyzed after 24 h of exposure. The focus was placed on the three lowest concentrations, where Ca‐, Sr‐, and Zn‐based materials promoted increased metabolic activity, whereas Ag‐based materials already showed signs of cytotoxicity. The results are summarized in Table 1, which presents the concentrations in micrograms per milliliter, while the corresponding values in micromolar and the percentage of ions leached relative to the initial amount added are provided in Tables S1 and S2, respectively. For instance, α‐Ag_2_WO_4_ released Ag^+^ up to 0.2644 μg/mL (2.45 μM) at 1.9 μg/mL, significantly higher than β‐Ag_2_MoO_4_ (0.1423 μg/mL [1.32 μM] at the same concentration). This aligns with the more severe cytotoxicity observed for α‐Ag_2_WO_4_, particularly under indirect exposure, where even the lowest doses produced unrecoverable metabolic suppression. In contrast, β‐Ag_2_MoO_4_ ionic release profile remained below critical thresholds, allowing for partial recovery of metabolic activity over time. It is also observed that β‐Ag_2_MoO_4_ exhibits total ionic leaching between 14% and 17% of its initial content, whereas for α‐Ag_2_WO_4_, this value ranges from 20% to 31%.

Regarding Zn‐based materials, interesting results emerge on the third day of exposure: β‐ZnMoO_4_ induces extremely high metabolic stimulation at all concentrations below 250 μg/mL, while ZnWO_4_ shows similar stimulation at all tested concentrations. Toxic levels for β‐ZnMoO_4_ are observed only above 250 μg/mL, revealing a fine threshold between toxicity and cellular stimulation. For Zn‐containing materials, Zn^2+^ release at lower concentrations remained below 1 μM (< 0.0617 μg/mL), which is well within the stimulatory range and may explain the pronounced metabolic activation seen at 72 h. A similar profile was observed for ZnWO_4_. Notably, these levels did not approach the toxicity threshold (~50 μM), supporting the conclusion that Zn^2+^ release at subtoxic levels contributes to cellular regeneration. The total ionic leaching for these materials remained between 1% and 4%, indicating greater stability in water compared with the Ag‐based materials.

With respect to Ca^2+^ and Sr^2+^, materials like CaMoO_4_ and SrMoO_4_ released up to 0.0925 (2.3 μM) and 0.0418 μg/mL (1.5 μM), respectively, at the highest dose analyzed (7.8 μg/mL). Similarly, CaWO_4_ and SrWO_4_ exhibited a little higher leaching behavior, with Ca^2+^ and Sr^2+^ levels also remaining within the low micromolar range. This total ionic leaching corresponded to approximately 1%–3% for these materials, except for CaWO_4_, for which the values ranged between 7% and 13%. At lower concentrations, all four materials released these ions at levels typically associated with bioactivity rather than toxicity. The slight metabolic stimulation observed at early time points may be attributed to this ionic contribution, especially considering that indirect contact experiments confirmed minimal ROS involvement for these systems.

For direct contact, ROS production, even in the dark, adds another layer of complexity. Some samples showed a reduction in metabolic activity on Day 3 compared with Day 1; however, this activity recovered by Day 7, suggesting that the cells underwent an adaptation period in response to the samples. At low concentrations, ROS function as signaling molecules, regulating essential biological processes such as cell proliferation, differentiation, and tissue repair (Dunnill et al. 2017). Furthermore, they can activate metabolic pathways that induce the expression of antioxidant and cytoprotective genes (Mathers et al. 2004), enhancing the cell's capacity to handle future damage. However, when ROS levels exceed physiological thresholds, they can damage DNA, proteins, and lipids, leading to cellular dysfunction and death via necrosis or apoptosis (Circu and Aw 2010; Ghosh et al. 2018). For Ag‐based materials (α‐Ag_2_WO_4_ and β‐Ag_2_MoO_4_), ROS production plays a key role in toxicity, with significant toxic levels observed even at low concentrations. These results align with previous studies investigating the cytotoxic properties of these materials in other cell lines, such as NIH/3T3 and THP‐1 (Fragelli et al. 2024; Pimentel et al. 2023).

For Ca‐based (CaMoO_4_ and CaWO_4_) and Sr‐based (SrMoO_4_ and SrWO_4_) materials, a slight reduction in metabolic activity is observed only at the highest concentrations, suggesting mildly toxic ROS generation at levels above 500 μg/mL. Interestingly, these materials also exhibit a stimulatory metabolic effect at lower concentrations, indicating that ROS production at these levels may be beneficial, as ionic release (confirmed via indirect contact) does not cause significant cellular stimulation. For Zn‐based materials, it is evident that ROS amplifies their toxicity at high concentrations (above 62.5 μg/mL). However, at longer exposure times and lower concentrations (below 62.5 μg/mL), ROS production, coupled with Zn^2+^ release, induces remarkably high levels of cellular stimulation.

#### 3.2.2 Intracellular ROS and RNS Production

**FIGURE 4:**
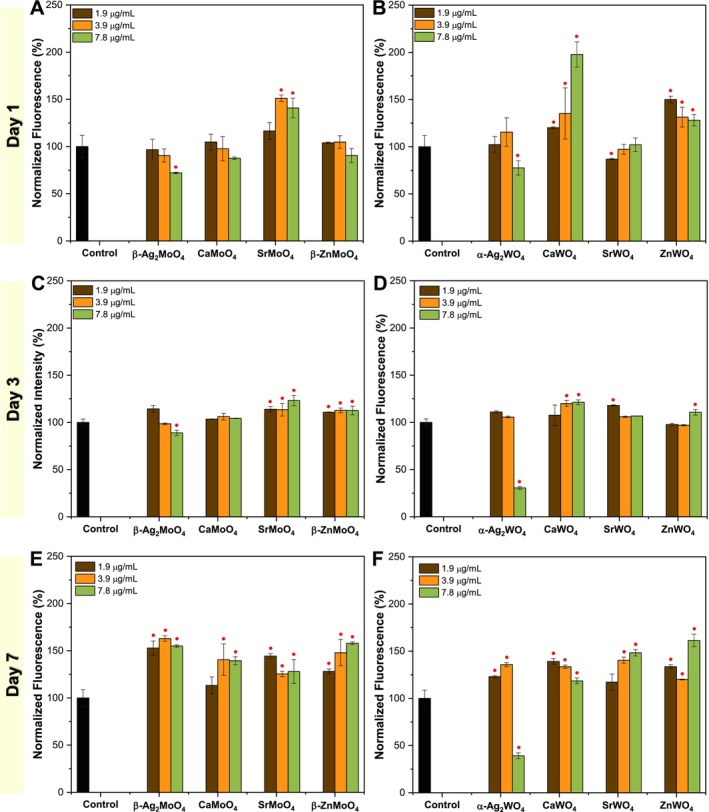
Intracellular ROS production via direct contact using L929 cells: evaluation of metal molybdates at (A) 1, (C) 3, and (E) 7 days, and metal tungstates at (B) 1, (D) 3, and (F) 7 days. (●/■) versus control: ● *p* ≤ 0.05; ■ *p* ≤ 0.01.

**FIGURE 5:**
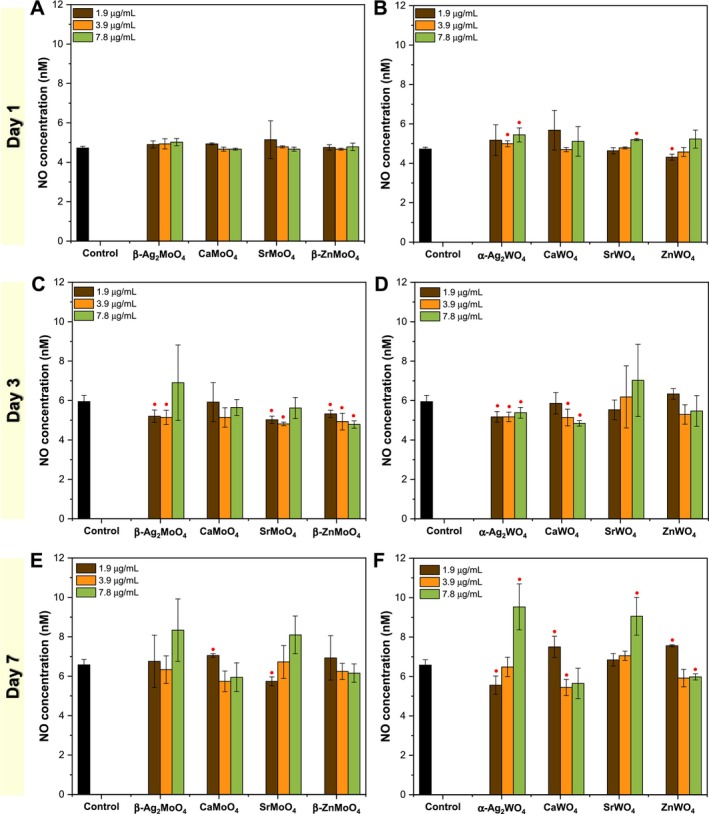
Intracellular RNS production via direct contact using L929 cells: evaluation of metal molybdates at (A) 1, (C) 3, and (E) 7 days, and metal tungstates at (B) 1, (D) 3, and (F) 7 days. (●/■) versus control: ● *p* ≤ 0.05; ■ *p* ≤ 0.01.

Cell metabolic activity tests reveal that L929 metabolism increases at lower concentrations of Ca‐, Sr‐, and Zn‐based materials, whereas Ag‐based materials exhibit toxic effects starting at 3.9 μg/mL. Consequently, ROS and RNS production were analyzed at concentrations of 1.9, 3.9, and 7.8 μg/mL, as shown in Figures 4 and 5. The increase in intracellular ROS production is generally a bad sign for cells, as it is closely linked to mitochondrial dysfunction (Fleury et al. 2002). When the electron transport chain becomes unstable, electrons escape and react with oxygen, forming superoxide radical (⦁O_2_^−^), a highly reactive molecule that can damage cellular components. On top of that, oxidative enzymes like NADPH oxidase and xanthine oxidase further contribute to excessive ROS generation, overwhelming the cell's natural antioxidant defenses (Bortolotti et al. 2021; Liu et al. 2021). To make matters worse, endoplasmic reticulum stress and lipid peroxidation set off a vicious cycle of cellular damage, further intensifying oxidative stress (Ashraf and Sheikh 2015). If left unchecked, this imbalance can lead to inflammation, DNA damage, and ultimately, cell death.

For molybdates, on the first day of exposure, only a slight but significant increase is observed for SrMoO_4_ at 3.9 and 7.8 μg/mL, while β‐Ag_2_MoO_4_ shows a slight reduction at the highest concentration. By the third day, SrMoO_4_ and β‐ZnMoO_4_ exhibit a moderate but significant increase in ROS production across all tested concentrations, whereas β‐Ag_2_MoO_4_ remains unchanged. The most prominent effect is observed on the seventh day, where all materials show a significant increase in ROS production at nearly all tested concentrations, except for CaMoO_4_ at 1.9 μg/mL. Regarding tungstates, on the first day, CaWO_4_ and ZnWO_4_ significantly increase ROS production, while SrWO_4_ at the lowest concentration and α‐Ag_2_WO_4_ at the highest concentration exhibit a slight reduction. By the third day, ROS levels remain close to the control, with minor variations in significance. However, α‐Ag_2_WO_4_ shows a sharp decline in ROS production at the highest concentration. On the seventh day, the trend aligns with that observed for molybdates, with a general increase in ROS production across all materials, except for the highest concentration of α‐Ag_2_WO_4_, which shows a reduction.

For Ag‐based materials, a notable decrease in ROS levels is observed, particularly at 7.8 μg/mL. This effect is attributed to the reduced viability of L929 cells when exposed to both β‐Ag_2_MoO_4_ and α‐Ag_2_WO_4_. In contrast, the intracellular ROS increase observed for Ca‐based materials may be linked to the activation of enzymes such as NADPH oxidase, which directly generates ROS (Görlach et al. 2015). Additionally, Ca^2+^ ions may overload mitochondria, leading to mitochondrial dysfunction and an increase in ROS due to disruptions in the electron transport chain (Gordeeva et al. 2003). Sr‐based materials exhibit a mechanism similar to Ca‐based ones, modulating pathways that affect mitochondrial metabolism and ROS production. Meanwhile, Zn‐based materials can disrupt cellular redox homeostasis by inhibiting antioxidant enzymes such as glutathione reductase (Dabravolski et al. 2022). Furthermore, extracellular ROS production by these particles may directly interact with unsaturated membrane lipids, triggering a cascade of lipid peroxidation (Adibhatla and Hatcher 2010).

Related to RNS, an excessive increase in nitric oxide (NO) inside cells can be harmful, especially when its production becomes uncontrolled. While NO plays a crucial role in cell signaling and vascular function, its overproduction can lead to cellular stress (C.‐N. Wang et al. 2015). High NO levels can interfere with normal cell function, damage proteins and lipids, and contribute to inflammation (Khansari et al. 2009). In some cases, NO reacts with other molecules, forming highly reactive compounds that further harm the cell. If this imbalance persists, it can disrupt metabolism and even trigger cell death, making proper NO regulation essential for maintaining healthy cellular activity. For the molybdates, no significant increase in intracellular RNS production is observed on the first day of exposure for any of the materials. By the third day, although the values remain close to control, a reduction in RNS levels is noted at the two lowest concentrations of β‐Ag_2_MoO_4_ and SrMoO_4_, as well as at all tested concentrations of β‐ZnMoO_4_. On the seventh day, despite greater variability in the data, a slight increase in RNS is detected at 1.9 μg/mL of CaMoO_4_, while a slight reduction occurs at the same concentration for SrMoO_4_. For the tungstates, minor changes are observed on the first day, but significant variations appear at 3.9 and 7.8 μg/mL of α‐Ag_2_WO_4_, at the highest concentration of SrWO_4_, and the lowest concentration of ZnWO_4_. By the third day, a slight reduction in RNS is seen across all concentrations of α‐Ag_2_WO_4_, as well as at 3.9 and 7.8 μg/mL of CaWO_4_. The most pronounced changes occur on the seventh day, where α‐Ag_2_WO_4_ and SrWO_4_ show a dose‐dependent increase in RNS, in contrast to CaWO_4_ and ZnWO_4_, which exhibit a dose‐dependent reduction.

The ions Ca^2+^, Sr^2+^, Zn^2+^, and Ag^+^, along with ROS, can influence RNS production by modulating nitric oxide synthase (NOS) activity and affecting cellular redox balance (Nabi et al. 2019). Ca^2+^ and Sr^2+^ activate this enzyme by enhancing calmodulin binding, leading to increased NO production, but excessive levels can cause enzyme dysfunction under oxidative stress (Schmidt et al. 1992). Zn^2+^ plays a structural role in NOS, but high concentrations may inhibit its activity, reducing NO levels (Perry et al. 2000). Ag^+^ can directly inhibit NOS by interacting with thiol groups or competing with essential cofactors (Jiang et al. 2019). Additionally, ROS can react with NO to form peroxynitrite (ONOO^−^), depleting NO bioavailability and contributing to oxidative and nitrosative stress (Aicardo et al. 2016). Interestingly, molybdate‐based materials appear to have little impact on RNS levels, possibly due to their lower interaction with NOS or their reduced ability to generate ROS that would react with NO. In contrast, tungstate‐based materials show a more pronounced effect disrupting NOS activity, while also promoting oxidative stress that alters NO metabolism. This suggests that tungstate‐based materials have a greater impact on nitrosative stress compared with molybdates.

A comparison between the intracellular ROS/RNS levels and the metabolic activity data (MTT) reveals important insights into the cellular response to the tested materials. For Ca‐, Sr‐, and Zn‐based samples, a moderate increase in ROS and, in some cases, RNS production is observed, particularly on Days 3 and 7. However, these increases coincide with maintained or even enhanced cell viability at the tested concentrations, suggesting that the oxidative imbalance induced by these materials may trigger adaptive responses rather than cytotoxicity. In contrast, Ag‐based materials show a distinct profile: despite a less pronounced ROS increase at certain time points, a sharp and early decline in metabolic activity is already evident from 3.9 μg/mL. This suggests that oxidative stress in these samples may reach critical thresholds more quickly, leading to mitochondrial dysfunction, impaired metabolism, and reduced cell numbers, factors that in turn influence the measurable levels of ROS and RNS. In this context, the lower ROS values observed for Ag‐containing samples at higher concentrations may reflect a loss of viable cells rather than reduced oxidative activity per se. These findings emphasize the importance of interpreting ROS/RNS levels in conjunction with viability assays, as cellular damage may either stimulate compensatory mechanisms or overwhelm the system depending on the material and concentration. Such integrated analysis offers a more accurate understanding of the oxidative and nitrosative stress dynamics in relation to cellular health.

#### 3.2.3 Apoptosis and Necrosis

To assess cell death mechanisms, apoptosis and necrosis were analyzed using EB and AO staining. This dual‐staining technique is widely used to differentiate between live, apoptotic, and necrotic cells based on their membrane integrity and nuclear morphology. Acridine orange is a permeable dye that stains all cells green by binding to nucleic acids, while ethidium bromide only penetrates cells with compromised membranes, staining their nuclei orange or red (Ude et al. 2022). This allows clear visualization of apoptotic cells, which exhibit chromatin condensation and nuclear fragmentation, and necrotic cells, which show diffuse staining due to loss of membrane integrity. Understanding whether a material induces apoptosis or necrosis is crucial, as apoptosis is a controlled, programmed cell death process, while necrosis often leads to inflammation and uncontrolled cell damage. This analysis provides valuable insights into the cytotoxic effects of the tested materials and their potential biocompatibility.

**FIGURE 6:**
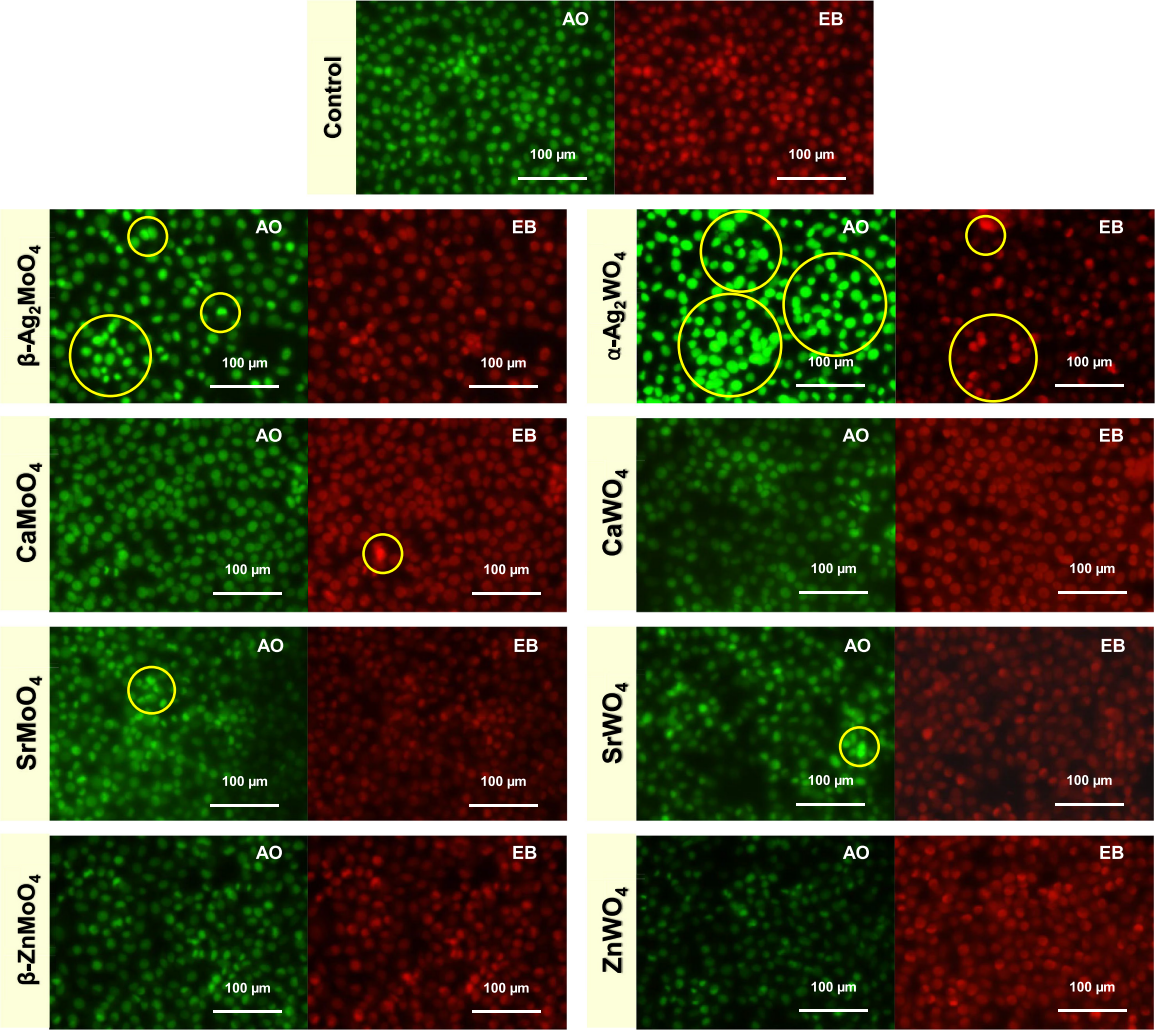
Apoptosis and necrosis analysis by fluorescence microscopy. Representative fluorescence images of L929 cells stained with AO and EB after 24 h of exposure to different materials at 3.9 μg/mL. Live cells appear green (AO‐positive, EB‐negative), apoptotic cells show bright green condensed or fragmented nuclei (AO‐positive), and necrotic cells appear orange/red due to loss of membrane integrity (EB‐positive).

These results are presented in Figure 6, where, as expected, only Ag‐based materials (α‐Ag_2_WO_4_ and β‐Ag_2_MoO_4_) induced apoptosis, with necrotic cells observed exclusively in α‐Ag_2_WO_4_‐treated samples. The analysis was performed at 3.9 μg/mL, a concentration where viable cells were still detected according to the MTT assay, even in the presence of Ag‐based materials. The ability of Ag^+^ ions and ROS to induce apoptosis and necrosis is well known, as Ag^+^ can interact with cellular thiol groups, disrupting redox homeostasis and triggering oxidative stress (Cortese‐Krott et al. 2009). ROS generation further damages lipids, proteins, and DNA, leading to mitochondrial dysfunction and activation of apoptotic pathways (Redza‐Dutordoir and Averill‐Bates 2016). In the case of α‐Ag_2_WO_4_, the presence of necrotic cells suggests that, beyond apoptosis, oxidative and nitrosative stress levels were high enough to cause membrane rupture and uncontrolled cell death. This may be related to the increase in both ROS and RNS, as previously observed in the analysis of oxidative stress markers. Importantly, this analysis was conducted only within the first 24 h, providing insights into the early‐stage cytotoxic effects of these materials. These findings suggest that the observed decrease in metabolic activity for Ag‐containing materials is not merely a transient adaptation but reflects early signs of irreversible damage. While ROS and RNS analyses provide important insights into oxidative stress, their increase, particularly in non‐Ag materials, did not correlate with reduced viability or cell death, likely indicating an adaptive cellular response. In contrast, in Ag‐based systems, the drop in metabolic activity aligns with the activation of cell death pathways, reinforcing the toxic potential of Ag^+^ ions and ROS production by the samples, even at concentrations where viability is not yet fully lost.

#### 3.2.4 Genotoxicity

Evaluating the genotoxic potential of materials is essential to ensure their safety, especially when they are designed for biomedical or environmental use. Genotoxic effects can result in alterations to DNA or chromosomes, potentially leading to long‐term biological consequences such as mutations or cancer (Turkez et al. 2017). A commonly used method for detecting such effects is the micronucleus assay using CHO‐K1 cells, which identifies the presence of small, extranuclear bodies formed from chromosomal fragments or entire chromosomes that fail to integrate into daughter nuclei during cell division (dos Santos Jorge Sousa et al. 2024). The appearance of micronuclei in cells is a clear sign of genetic damage, making this test a practical and sensitive tool for identifying harmful interactions at the genetic level.

**FIGURE 7:**
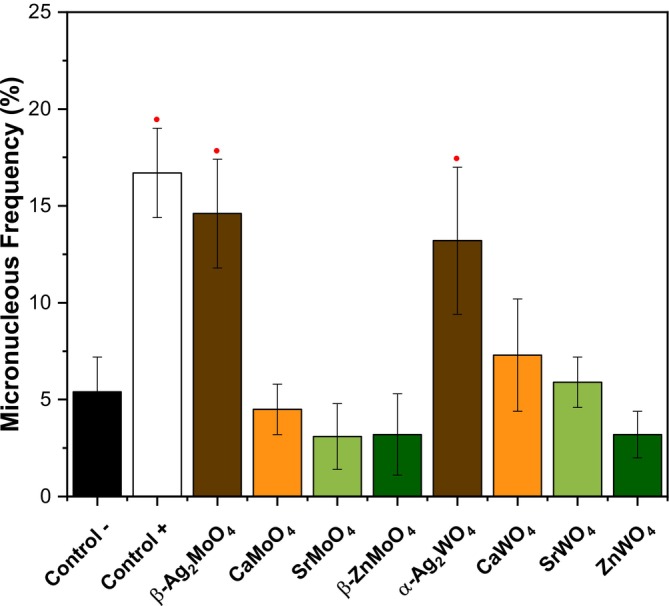
Results of micronucleus assay of CHO‐K1 cell line during the experimental period of 24 h. (●/■) versus control: ● *p* ≤ 0.05; ■ *p* ≤ 0.01. The results were presented as the median with the upper and lower quartiles: Me [Q1; Q3].

In this context, the micronucleus assay was performed after 24 h of exposure to the particles at a concentration of 3.9 μg/mL, a condition under which Ag‐based materials showed the lowest cytotoxic effects (Figure 7). Additionally, at this concentration, L929 cells displayed increased metabolic activity when exposed to the other materials. For the assay, the negative control consisted of untreated cell cultures, while the positive control was established using MMS. As expected, a baseline incidence of micronuclei was observed in the negative control, with frequencies exceeding 5%. In contrast, the positive control with MMS resulted in a marked increase, reaching frequencies close to 17%.

For the tested materials, the results suggest that the lattice former ([MoO_4_]^2−^ or [WO_4_]^2−^) is not the determining factor in micronucleus formation. Instead, the lattice modifier appears to play a more critical role. Among the Ag‐based materials, although no significant cytotoxicity was observed, a statistically relevant increase in micronucleus frequency was detected, comparable to the positive control, indicating a genotoxic response. The observed increase in micronucleus formation among Ag‐based materials may be linked to the predominance of apoptosis as the main cell death mechanism, given that necrosis was not detected in β‐Ag_2_MoO_4_. Since apoptosis is a regulated process that allows the cell to progress through DNA damage responses before complete loss of viability (Elmore 2007), it may create favorable conditions for chromosomal alterations to be expressed as micronuclei. These findings differ from those reported by Chaves et al., who observed no loss of genomic DNA integrity in HGF cells exposed to α‐Ag_2_WO_4_ particles at concentrations ranging from 0.781 to 78.1 μg/mL (Haro Chávez et al. 2018). This discrepancy may be explained by the fact that some materials do not induce DNA strand breaks detectable by conventional assays, but may still interfere with chromosomal segregation or DNA repair mechanisms, ultimately leading to micronucleus formation. As for the remaining materials tested, none showed a significant increase in micronucleus frequency, suggesting a likely safety profile for their potential biological applications.

The biological responses observed in this study provide important insights into the potential application of these materials in the biomedical field. The favorable behavior of Ca‐, Sr‐, and Zn‐based tungstates and molybdates, particularly their ability to stimulate metabolic activity at lower concentrations without inducing significant genotoxic effects, highlights their suitability as candidates for the development of safe and functional biomaterials. These elements are already known to play beneficial roles in bone regeneration, cellular proliferation, and tissue integration, reinforcing their relevance for use in areas such as tissue engineering, bone graft substitutes, and antimicrobial coatings. In contrast, while Ag‐based materials demonstrated strong biological activity, including antimicrobial potential, they also exhibited early signs of cytotoxicity and, more critically, induced genotoxic effects at noncytotoxic doses. The increase in micronucleus formation, along with apoptosis and necrosis at low concentrations, raises concerns regarding the long‐term safety of Ag‐containing compounds. Therefore, although Ag‐based tungstates and molybdates may be useful in short‐term or localized applications where antimicrobial action is essential, their use in long‐term or systemic biomedical devices should be approached with caution. Further studies, including in vivo evaluation and long‐term exposure analysis, are necessary to fully determine their applicability in clinical settings.

Taking together the use of multiple biomarkers, such as metabolic activity, apoptosis, necrosis, and micronucleus formation, enables a more integrated understanding of the biological risks posed by these materials. This combined analysis helps differentiate compounds that are primarily cytotoxic from those that exert genotoxic effects even at subcytotoxic concentrations. Nonetheless, it is important to recognize the methodological limitations of each assay, including variability in sensitivity, specificity, and relevance to in vivo outcomes. Therefore, while the current findings provide a robust initial safety assessment, further validation through complementary models remains essential.

## 4 Conclusions

In conclusion, this study successfully synthesized a series of transition metal tungstates and molybdates, allowing for a comprehensive evaluation of their behavior both in solid state and in solution. Cytotoxicity assays conducted over 1, 3, and 7 days revealed that Ag‐based materials exhibited toxicity at low concentrations toward L929 cells. In contrast, Ca‐ and Sr‐based materials could stimulate cellular metabolic activity at lower doses. Zn‐based compounds also promoted metabolic activity at low concentrations, though toxicity was observed at levels above 62.5 μg/mL. In all cases, cytotoxic effects were accompanied by noticeable changes in cell morphology.

Importantly, the main mechanism behind the observed toxicity appears to be oxidative stress, particularly through ROS generation. Indirect assays showed minimal toxicity except in Ag‐based materials, suggesting that ROS production plays a central role in their biological effects. Intracellular oxidative stress was confirmed by quantifying ROS and RNS levels. A mild increase in intracellular ROS was noted on Day 1 for transition metal molybdates and became more evident by Day 7 for all materials. RNS levels, on the other hand, were significantly elevated on Day 7, especially for α‐Ag_2_WO_4_ and SrWO_4_ at higher concentrations.

The analysis of oxidative stress biomarkers (ROS/RNS) in conjunction with the MTT assay revealed that Ca‐, Sr‐, and Zn‐based materials induced moderate oxidative changes while maintaining or even enhancing cell viability, suggesting a cellular adaptation process. In contrast, Ag‐based materials triggered a sharp reduction in metabolic activity even at low concentrations, aligning with early mitochondrial dysfunction and the activation of cell death pathways. These differences reinforce that oxidative stress alone does not dictate toxicity outcomes; rather, the cellular capacity for adaptation plays a critical role.

The type of cell death further supports this distinction. At 3.9 μg/mL, both Ag‐based materials induced apoptosis, while α‐Ag_2_WO_4_ also triggered necrosis, a more abrupt form of cell death. This progression from regulated to uncontrolled cell death aligns with the severity of the cytotoxic response observed in MTT and AO/EB assays. Notably, micronucleus formation, used as an indicator of genotoxicity, was significantly increased only in Ag‐based materials, with β‐Ag_2_MoO_4_ showing the most pronounced effect despite the absence of necrosis. Since apoptosis is a regulated process that allows cells to pass through DNA damage checkpoints, it may favor the manifestation of chromosomal alterations as micronuclei. In contrast, necrotic cells may undergo rapid lysis before nuclear damage can be fully expressed.

Altogether, these findings demonstrate that the use of complementary biomarkers, encompassing oxidative stress, metabolic activity, cell death mechanisms, and chromosomal damage, enables a more nuanced and reliable assessment of the biological impact of new materials. This integrative approach not only differentiates between cytotoxic and genotoxic effects but also highlights the necessity of using multi‐parametric panels in the safety evaluation of nanomaterials. Ca‐, Sr‐, and Zn‐based tungstates and molybdates appear to be safe at the tested concentrations and show promising potential for biomedical and environmental applications. In contrast, Ag‐based materials, despite their functional properties, require further investigation due to their dual cytotoxic and genotoxic behavior, particularly in long‐term exposure contexts.

## Conflicts of Interest

The authors declare no conflicts of interest.

## Supporting information


**Table S1.** Ionic concentrations (μM) released into the medium after 24 h of exposure for the different materials, measured by ICP analysis at the three lowest tested concentrations.
**Table S2.** Percentage of total ionic leaching relative to the initial ion content for the different materials after 24 h of exposure, measured by ICP analysis at the three lowest tested concentrations.

**Figure S1.** Hydrodynamic size of the samples.
**Figure S2.** Zeta potential of the samples.
**Figure S3.** Cell metabolic activity using MTT assay via indirect contact using L929 cells: evaluation of metal molybdates at **A)** 1, **C)** 3, and **E)** 7 days, and metal tungstates at **B)** 1, **D)** 3, and **F)** 7 days. (●/■) vs Control: ● p ≤ 0.05; ■ p ≤ 0.01.
**Figure S4.** Cell metabolic activity via the MTT assay and optical microscopy in L929 cells exposed to β‐Ag2MoO4 at **A)** day 1, **B)** day 3, and **C)** day 7 under direct contact conditions. (●/■) vs Control: ● *p* ≤ 0.05; ■ *p* ≤ 0.01.
**Figure S5.** Cell metabolic activity via the MTT assay and optical microscopy in L929 cells exposed to β‐Ag2MoO4 at **A)** day 1, **B)** day 3, and **C)** day 7 under indirect contact conditions. (●/■) vs Control: ● *p* ≤ 0.05; ■ *p* ≤ 0.01.
**Figure S6.** Cell metabolic activity via the MTT assay and optical microscopy in L929 cells exposed to α‐Ag2WO4 at **A)** day 1, **B)** day 3, and **C)** day 7 under direct contact conditions. (●/■) vs Control: ● *p* ≤ 0.05; ■ *p* ≤ 0.01.
**Figure S7.** Cell metabolic activity via the MTT assay and optical microscopy in L929 cells exposed to α‐Ag2WO4 at **A)** day 1, **B)** day 3, and **C)** day 7 under indirect contact conditions. (●/■) vs Control: ● *p* ≤ 0.05; ■ *p* ≤ 0.01.
**Figure S8.** Cell metabolic activity via the MTT assay and optical microscopy in L929 cells exposed CaMoO4 at **A)** day 1, **B)** day 3, and **C)** day 7 under direct contact conditions. (●/■) vs Control: ● *p* ≤ 0.05; ■ *p* ≤ 0.01.
**Figure S9.** Cell metabolic activity via the MTT assay and optical microscopy in L929 cells exposed CaMoO4 at **A)** day 1, **B)** day 3, and **C)** day 7 under indirect contact conditions. (●/■) vs Control: ● *p* ≤ 0.05; ■ *p* ≤ 0.01.
**Figure S9.** Cell metabolic activity via the MTT assay and optical microscopy in L929 cells exposed CaMoO4 at **A)** day 1, **B)** day 3, and **C)** day 7 under indirect contact conditions. (●/■) vs Control: ● *p* ≤ 0.05; ■ *p* ≤ 0.01.
**Figure S10.** Cell metabolic activity via the MTT assay and optical microscopy in L929 cells exposed CaWO4 at **A)** day 1, **B)** day 3, and **C)** day 7 under direct contact conditions. (●/■) vs Control: ● *p* ≤ 0.05; ■ *p* ≤ 0.01.
**Figure S11.** Cell metabolic activity via the MTT assay and optical microscopy in L929 cells exposed CaWO4 at **A)** day 1, **B)** day 3, and **C)** day 7 under indirect contact conditions. (●/■) vs Control: ● *p* ≤ 0.05; ■ *p* ≤ 0.01.
**Figure S12.** Cell metabolic activity assessed via the MTT assay and optical microscopy in L929 cells exposed SrMoO4 at **A)** day 1, **B)** day 3, and **C)** day 7 under direct contact conditions. (●/■) vs Control: ● p 2 ≤ 0.05; ■ *p* ≤ 0.01.
**Figure S13.** Cell metabolic activity via the MTT assay and optical microscopy in L929 cells exposed SrMoO4 at **A)** day 1, **B)** day 3, and **C)** day 7 under indirect contact conditions. (●/■) vs Control: ● *p* ≤ 0.05; ■ *p* ≤ 0.01.
**Figure S14.** Cell metabolic activity via the MTT assay and optical microscopy in L929 cells exposed SrWO4 at **A)** day 1, **B)** day 3, and **C)** day 7 under direct contact conditions. (●/■) vs Control: ● *p* ≤ 0.05; ■ *p* ≤ 0.01.
**Figure S15.** Cell metabolic activity via the MTT assay and optical microscopy in L929 cells exposed SrWO4 at **A)** day 1, **B)** day 3, and **C)** day 7 under indirect contact conditions. (●/■) vs Control: ● *p* ≤ 0.05; ■ *p* ≤ 0.01.
**Figure S16.** Cell metabolic activity via the MTT assay and optical microscopy in L929 cells exposed β‐ZnMoO4 at **A)** day 1, **B)** day 3, and **C)** day 7 under direct contact conditions. (●/■) vs Control: ● *p* ≤ 0.05; ■ *p* ≤ 0.01.
**Figure S17.** Cell metabolic activity via the MTT assay and optical microscopy in L929 cells exposed β‐ ZnMoO4 at **A)** day 1, **B)** day 3, and **C)** day 7 under indirect contact conditions. (●/■) vs Control: ● *p* ≤ 0.05; ■ *p* ≤ 0.01.
**Figure S18.** Cell metabolic activity via the MTT assay and optical microscopy in L929 cells exposed ZnWO4 at **A)** day 1, **B)** day 3, and **C)** day 7 under direct contact conditions. (●/■) vs Control: ● *p* ≤ 0.05; ■ *p* ≤ 0.01.
**Figure S19** Cell metabolic activity via the MTT assay and optical microscopy in L929 cells exposed ZnWO4 at **A)** day 1, **B)** day 3, and **C)** day 7 under indirect contact conditions. (●/■) vs Control: ● *p* ≤ 0.05; ■ p ≤ 0.01.
**Figure S20.** pH time evolution of DMEM cell medium with **A)** transition metal molybdites and **B)** 1 transition metal tungstates. 2

## Data Availability

The data that support the findings of this study are available from the corresponding author upon reasonable request.
