# Supplementary material for: Deciphering the Toxicity of Metal Tungstates and Molybdates: Effects on L929 Cell Metabolic Activity, Oxidative Stress, and Genotoxicity
Source: J Appl Toxicol. 2025 Jun 22;45(10):2197–216. doi: 10.1002/jat.4836 (PMC12371800; doi:10.1002/jat.4836)
Supplement: Supplementary file 1 — Table S1. Ionic concentrations (μM) released into the medium after 24 h of exposure for the different materials, measured by ICP analysis at the three lowest tested concentrations. Table S2. Percentage of total ionic leaching relative to the initial ion content for the different materials after 24 h of exposure, measured by ICP analysis at the three lowest tested concentrations. [file JAT-45-2197-s001.docx]

**Table S1**. Ionic concentrations (µM) released into the medium after 24 hours of exposure for the different materials, measured by ICP analysis at the three lowest tested concentrations.

| **Concentration (µg/mL)** | **β-Ag_2_MoO_4_** | | **CaMoO_4_** | | **SrMoO_4_** | | **β-ZnMoO_4_** | |
| --- | --- | --- | --- | --- | --- | --- | --- | --- |
|  | **Ag (µM)** | **Mo (µM)** | **Ca (µM)** | **Mo (µM)** | **Sr (µM)** | **Mo (µM)** | **Zn (µM)** | **Mo (µM)** |
| **1.9** | 1.32 | 1.93 | 0.53 | 0.41 | 0.32 | 0.18 | 0.38 | 0.37 |
| **3.9** | 2.15 | 4.34 | 1.30 | 0.90 | 0.45 | 0.45 | 0.56 | 0.65 |
| **7.8** | 3.69 | 7.90 | 2.31 | 1.31 | 1.49 | 0.73 | 0.82 | 0.86 |
| **Concentration (µg/mL)** | **α-Ag_2_WO_4_** | | **CaWO_4_** | | **SrWO_4_** | | **ZnWO_4_** | |
|  | **Ag (µM)** | **W (µM)** | **Ca (µM)** | **W (µM)** | **Sr (µM)** | **W (µM)** | **Zn (µM)** | **W (µM)** |
| **1.9** | 2.45 | 1.79 | 2.46 | 0.80 | 0.70 | 0.21 | 0.48 | 0.19 |
| **3.9** | 2.95 | 4.09 | 3.56 | 1.50 | 0.94 | 0.30 | 0.65 | 0.42 |
| **7.8** | 5.04 | 5.84 | 5.02 | 2.16 | 1.42 | 0.39 | 0.94 | 0.53 |

**Table S2.** Percentage of total ionic leaching relative to the initial ion content for the different materials after 24 hours of exposure, measured by ICP analysis at the three lowest tested concentrations.

| **Inicial Concentration (µg/mL)** | **β-Ag_2_MoO_4_** | | | **CaMoO_4_** | | | **SrMoO_4_** | | | **β-ZnMoO_4_** | | |
| --- | --- | --- | --- | --- | --- | --- | --- | --- | --- | --- | --- | --- |
|  | **Ag**  **(% w/w)** | **Mo**  **(% w/w)** | **Total**  **(% w/w)** | **Ca**  **(% w/w)** | **Mo**  **(% w/w)** | **Total**  **(% w/w)** | **Sr**  **(% w/w)** | **Mo**  **(% w/w)** | **Total**  **(% w/w)** | **Zn**  **(% w/w)** | **Mo**  **(% w/w)** | **Total**  **(% w/w)** |
| **1.9** | 7.49 | 9.77 | 17.26 | 1.12 | 2.09 | 3.21 | 0.47 | 0.89 | 1.36 | 1.31 | 1.87 | 3.18 |
| **3.9** | 5.95 | 10.67 | 16.63 | 1.34 | 2.20 | 3.54 | 0.33 | 1.10 | 1.42 | 0.94 | 1.61 | 2.55 |
| **7.8** | 5.11 | 9.72 | 14.83 | 1.19 | 1.61 | 2.79 | 0.54 | 0.89 | 1.43 | 0.68 | 1.05 | 1.74 |
| **Concentration (µg/mL)** | **α-Ag_2_WO_4_** | | | **CaWO_4_** | | | **SrWO_4_** | | | **ZnWO_4_** | | |
|  | **Ag**  **(% w/w)** | **W**  **(% w/w)** | **Total**  **(% w/w)** | **Ca**  **(% w/w)** | **W**  **(% w/w)** | **Total**  **(% w/w)** | **Sr**  **(% w/w)** | **W**  **(% w/w)** | **Total**  **(% w/w)** | **Zn**  **(% w/w)** | **W**  **(% w/w)** | **Total**  **(% w/w)** |
| **1.9** | 13.92 | 17.31 | 31.23 | 5.19 | 7.73 | 12.92 | 1.04 | 2.05 | 3.09 | 1.64 | 1.87 | 3.52 |
| **3.9** | 8.15 | 19.30 | 27.45 | 3.66 | 7.05 | 10.71 | 0.68 | 1.41 | 2.08 | 1.09 | 1.98 | 3.07 |
| **7.8** | 6.97 | 13.76 | 20.73 | 2.58 | 5.09 | 7.67 | 0.51 | 0.93 | 1.44 | 0.79 | 1.25 | 2.04 |
