# Supplementary material for: Deciphering the Toxicity of Metal Tungstates and Molybdates: Effects on L929 Cell Metabolic Activity, Oxidative Stress, and Genotoxicity
Source: J Appl Toxicol. 2025 Jun 22;45(10):2197–216. doi: 10.1002/jat.4836 (PMC12371800; doi:10.1002/jat.4836)
Supplement: Supplementary file 2 — Figure S1. Hydrodynamic size of the samples. Figure S2. Zeta potential of the samples. Figure S3. Cell metabolic activity using MTT assay via indirect contact using L929 cells: evaluation of metal molybdates at A) 1, C) 3, and E) 7 days, and metal tungstates at B) 1, D) 3, and F) 7 days. (●/■) vs Control: ● p ≤ 0.05; ■ p ≤ 0.01. Figure S4. Cell metabolic activity via the MTT assay and optical microscopy in L929 cells exposed to β‐Ag2MoO4 at A) day 1, B) day 3, and C) day 7 under direct contact conditions. (●/■) vs Control: ● p ≤ 0.05; ■ p ≤ 0.01. Figure S5. Cell metabolic activity via the MTT assay and optical microscopy in L929 cells exposed to β‐Ag2MoO4 at A) day 1, B) day 3, and C) day 7 under indirect contact conditions. (●/■) vs Control: ● p ≤ 0.05; ■ p ≤ 0.01. Figure S6. Cell metabolic activity via the MTT assay and optical microscopy in L929 cells exposed to α‐Ag2WO4 at A) day 1, B) day 3, and C) day 7 under direct contact conditions. (●/■) vs Control: ● p ≤ 0.05; ■ p ≤ 0.01. Figure S7. Cell metabolic activity via the MTT assay and optical microscopy in L929 cells exposed to α‐Ag2WO4 at A) day 1, B) day 3, and C) day 7 under indirect contact conditions. (●/■) vs Control: ● p ≤ 0.05; ■ p ≤ 0.01. Figure S8. Cell metabolic activity via the MTT assay and optical microscopy in L929 cells exposed CaMoO4 at A) day 1, B) day 3, and C) day 7 under direct contact conditions. (●/■) vs Control: ● p ≤ 0.05; ■ p ≤ 0.01. Figure S9. Cell metabolic activity via the MTT assay and optical microscopy in L929 cells exposed CaMoO4 at A) day 1, B) day 3, and C) day 7 under indirect contact conditions. (●/■) vs Control: ● p ≤ 0.05; ■ p ≤ 0.01. Figure S9. Cell metabolic activity via the MTT assay and optical microscopy in L929 cells exposed CaMoO4 at A) day 1, B) day 3, and C) day 7 under indirect contact conditions. (●/■) vs Control: ● p ≤ 0.05; ■ p ≤ 0.01. Figure S10. Cell metabolic activity via the MTT assay and optical microscopy in L929 cells exposed CaWO4 at A) day 1, B) day 3, [file JAT-45-2197-s002.pdf]

Deciphering the Toxicity of Metal Tungstates and Molybdates: Effects on L929 Cell Metabolic Activity, Oxidative Stress, and Genotoxicity

M. Assis[1,*], A. de Souza[1], K.S.J. Sousa[1], D.G.N. Nina[1], M. Bonfacio[1], R. N. Granito[1], A.C.M. Rennó[1]

[1]Department of Biosciences, Federal University of São Paulo (UNIFESP), Santos, SP, Brazil.

* Corresponding author: marcelostassis@gmail.com

SUPPORTING INFORMATION

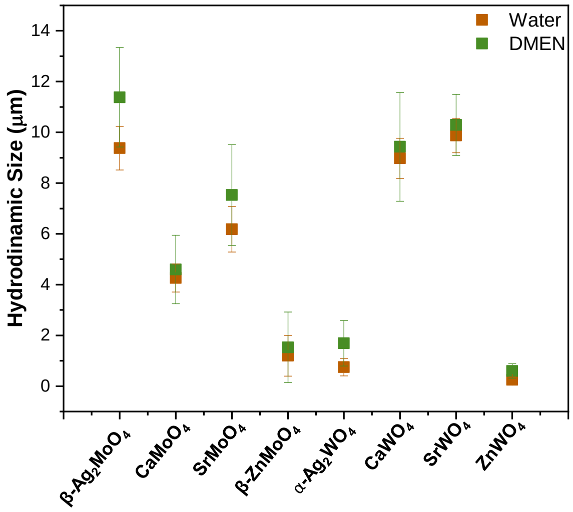

Figure S1. Hydrodynamic size of the samples.

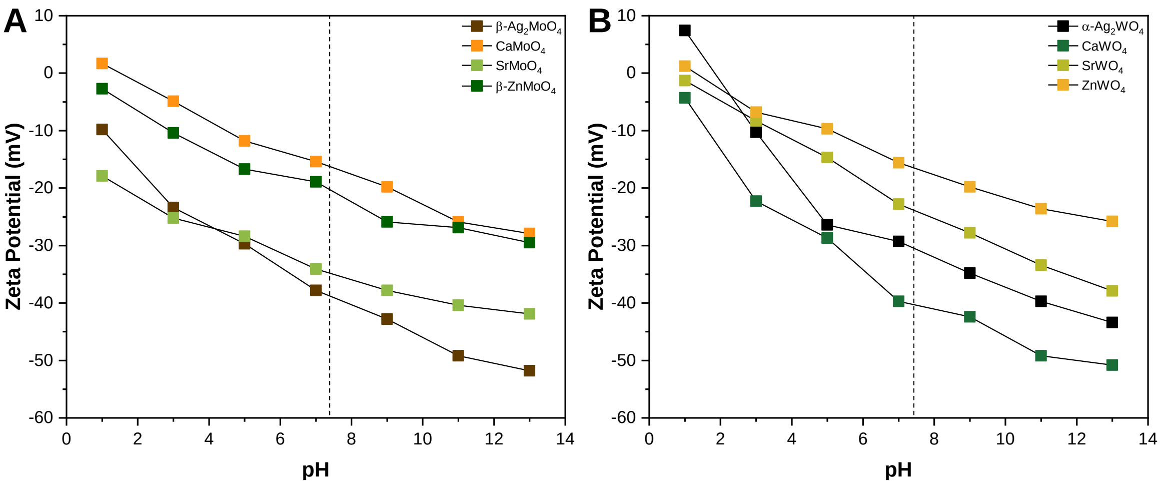

Figure S2. Zeta potential of the samples.

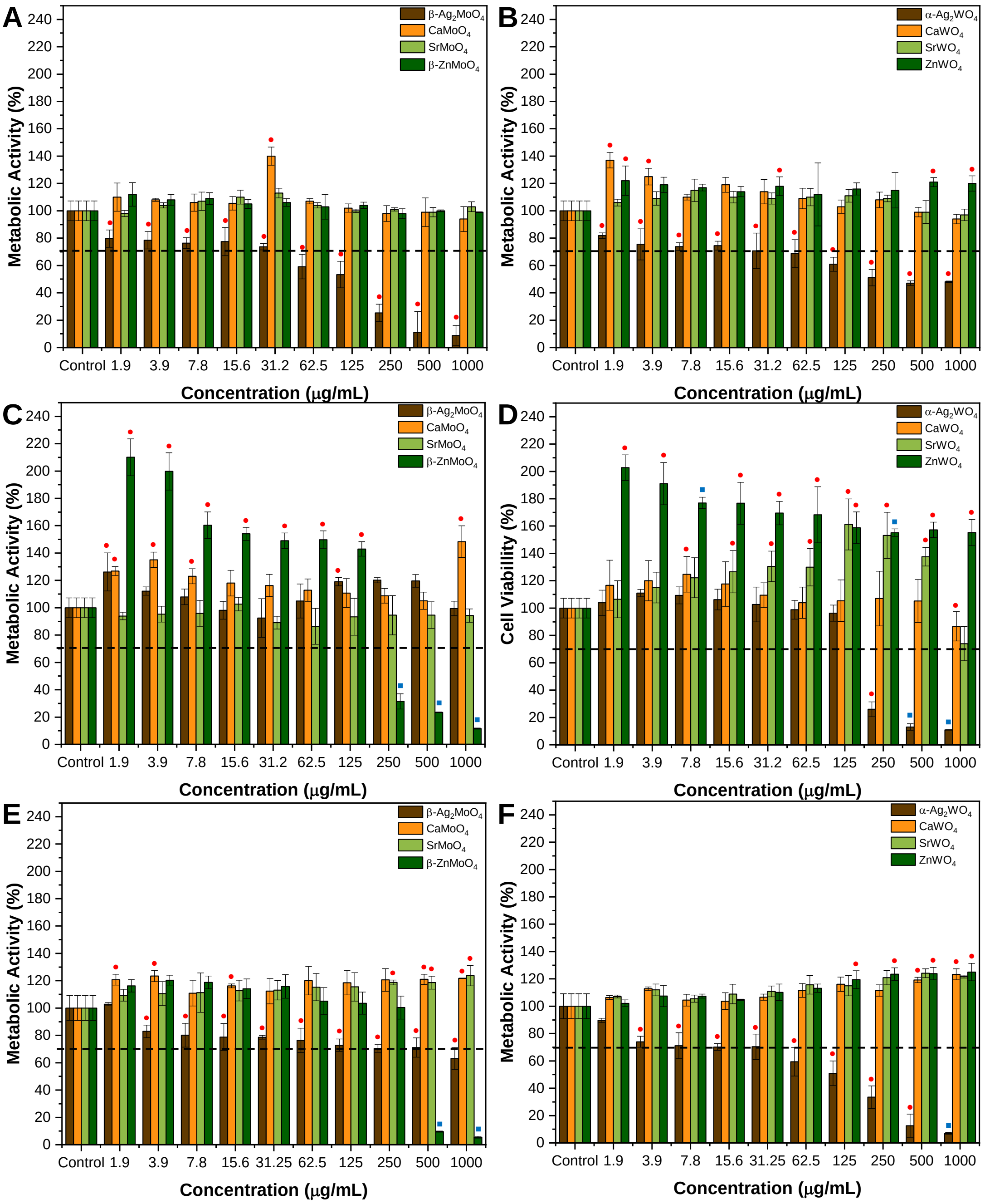

Figure S3. Cell metabolic activity using MTT assay via indirect contact using L929 cells: evaluation of metal molybdates at **A)** 1, **C)** 3, and **E)** 7 days, and metal tungstates at **B)** 1, **D)** 3, and **F)** 7 days. (●/■) vs Control: ● $p \leq 0.05$; ■ $p \leq 0.01$.

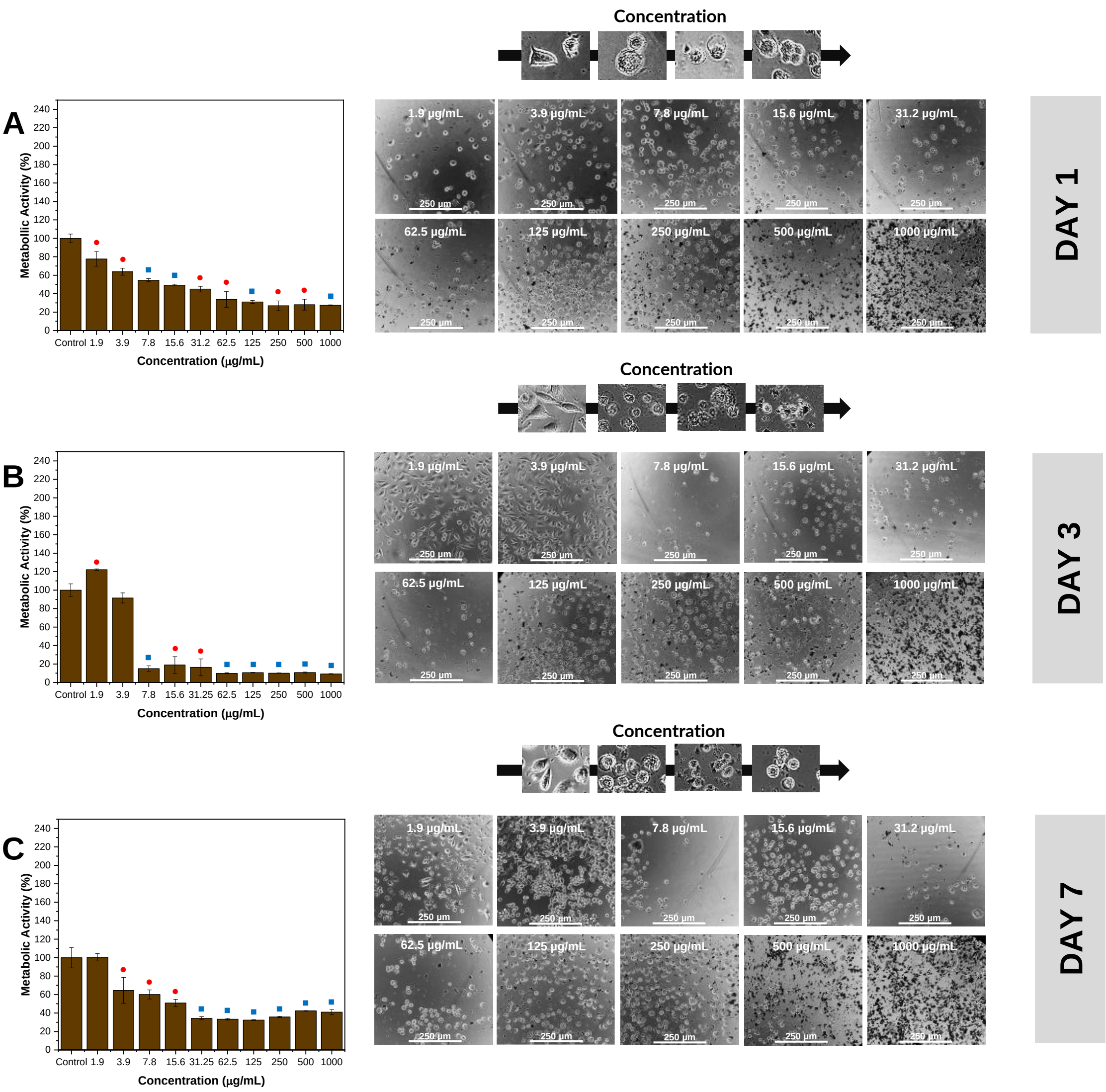

Figure S4. Cell metabolic activity via the MTT assay and optical microscopy in L929 cells exposed to β-Ag_2MoO_4 at **A)** day 1, **B)** day 3, and **C)** day 7 under direct contact conditions. (●/■) vs Control: ● $p \leq 0.05$; ■ $p \leq 0.01$.

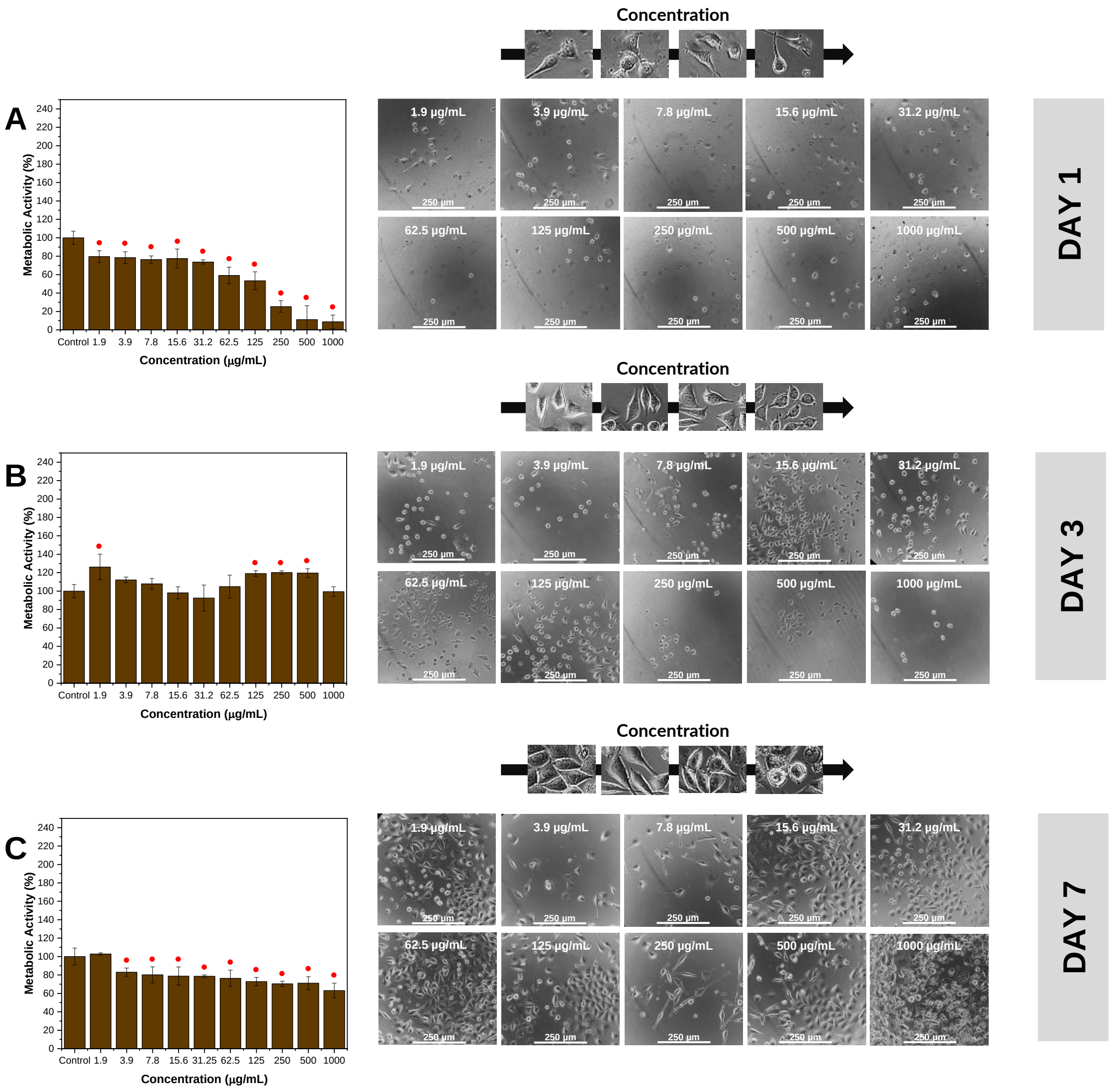

Figure S5. Cell metabolic activity via the MTT assay and optical microscopy in L929 cells exposed to β-Ag_2MoO_4 at **A)** day 1, **B)** day 3, and **C)** day 7 under indirect contact conditions. (●/■) vs Control: ● $p \leq 0.05$; ■ $p \leq 0.01$.

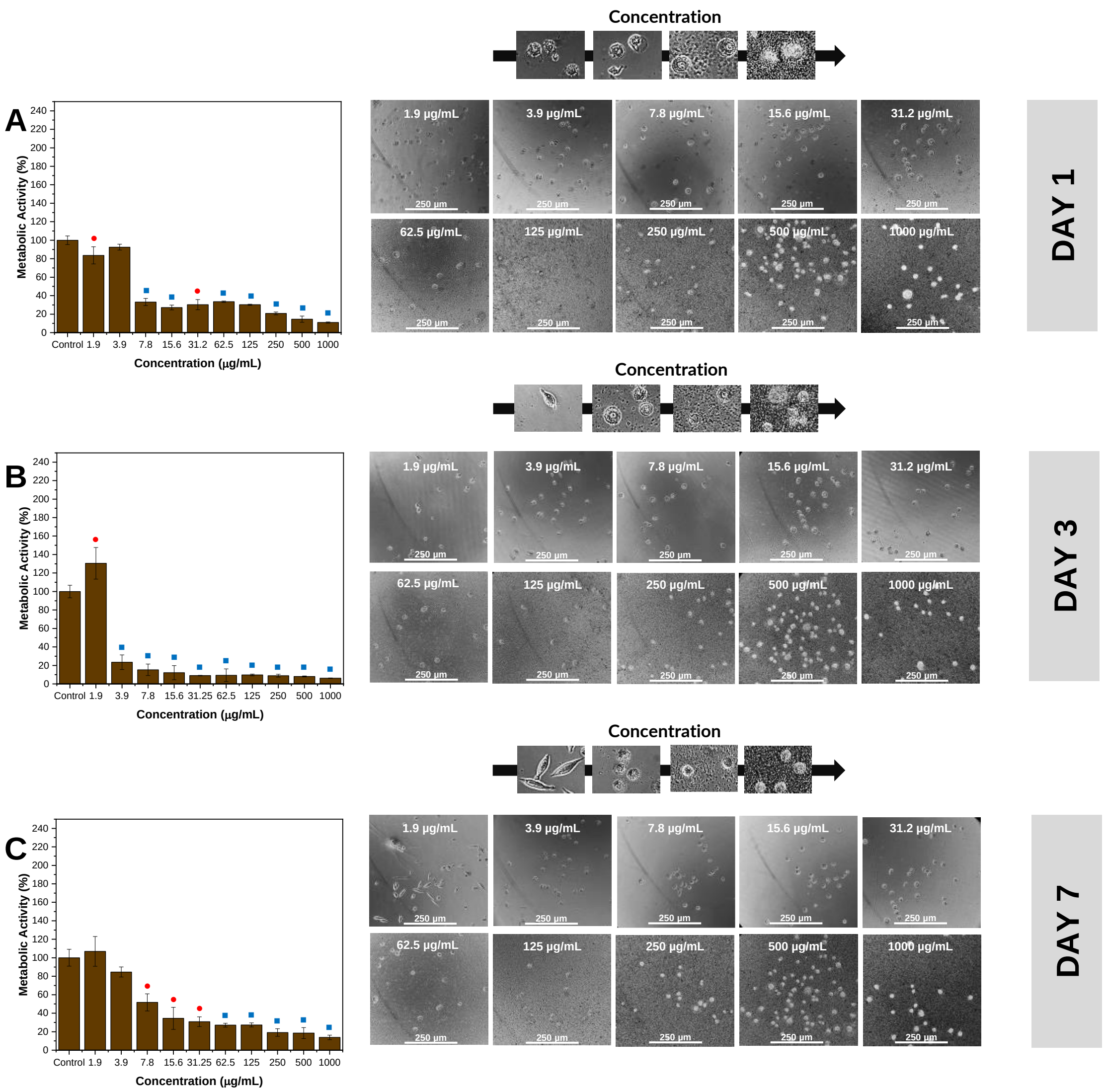

1 **Figure S6.** Cell metabolic activity via the MTT assay and optical microscopy in L929 cells exposed to α-
2 Ag_2WO_4 at **A)** day 1, **B)** day 3, and **C)** day 7 under direct contact conditions. (●/■) vs Control: ● $p \leq 0.05$;
3 ■ $p \leq 0.01$.
4

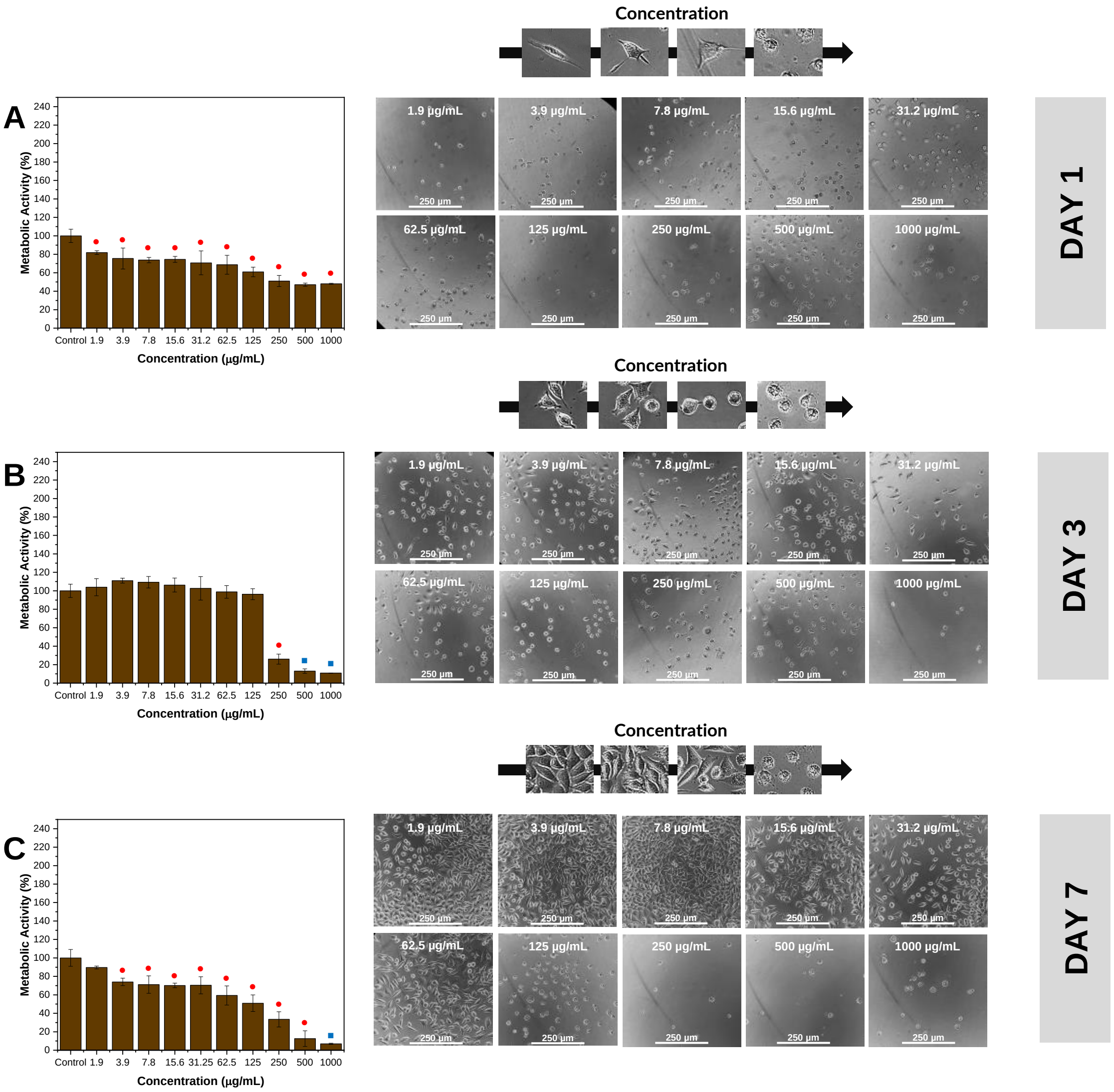

Figure S7. Cell metabolic activity via the MTT assay and optical microscopy in L929 cells exposed to α-Ag_2WO_4 at **A)** day 1, **B)** day 3, and **C)** day 7 under indirect contact conditions. (●/■) vs Control: ● $p \leq 0.05$; ■ $p \leq 0.01$.

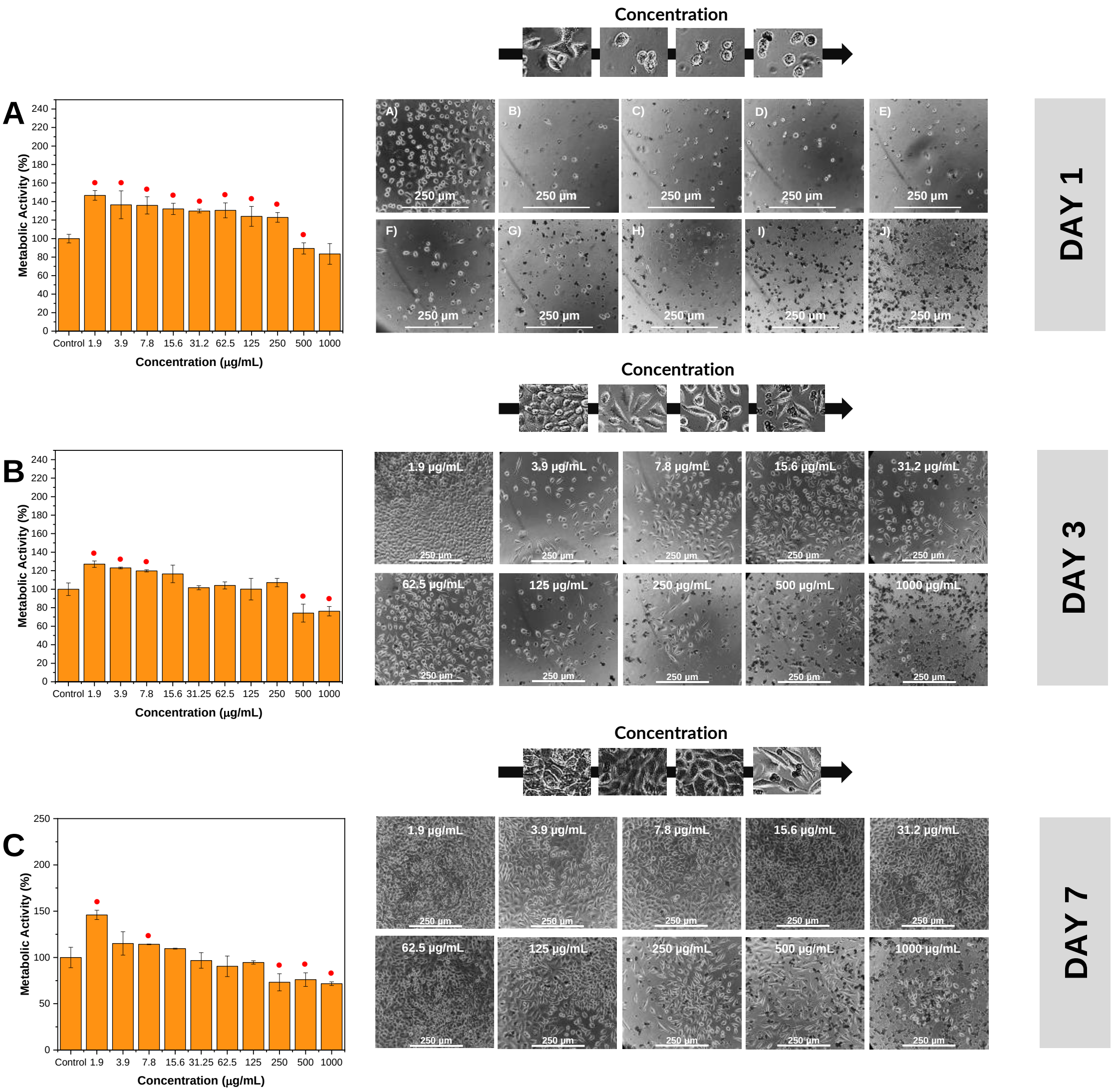

Figure S8. Cell metabolic activity via the MTT assay and optical microscopy in L929 cells exposed $CaMoO_4$ at **A)** day 1, **B)** day 3, and **C)** day 7 under direct contact conditions. (●/■) vs Control: ● $p \leq 0.05$; ■ $p \leq 0.01$.

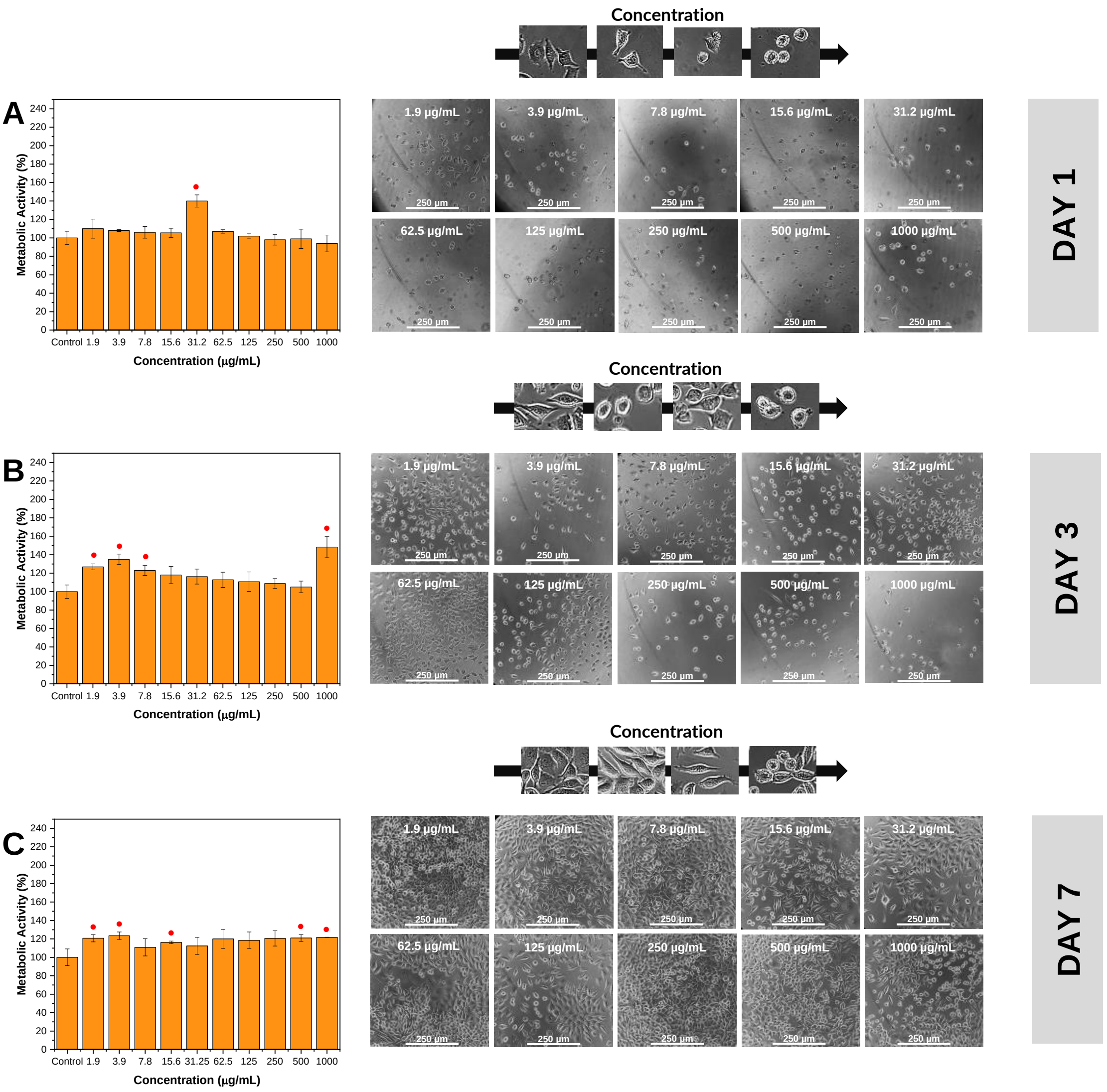

Figure S9. Cell metabolic activity via the MTT assay and optical microscopy in L929 cells exposed $CaMoO_4$ at **A)** day 1, **B)** day 3, and **C)** day 7 under indirect contact conditions. (●/■) vs Control: ● $p \leq 0.05$; ■ $p \leq 0.01$.

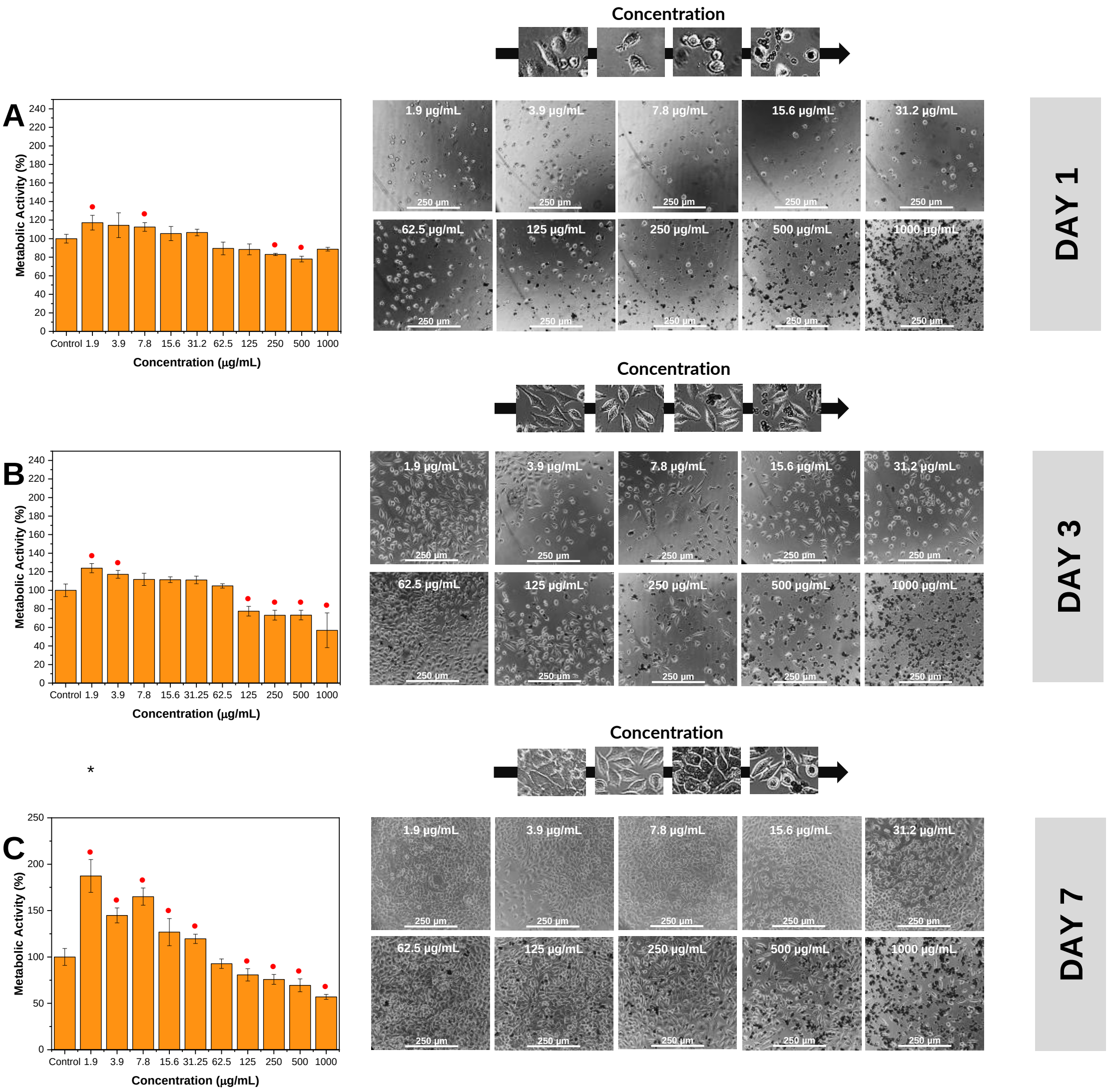

Figure S10. Cell metabolic activity via the MTT assay and optical microscopy in L929 cells exposed $CaWO_4$ at **A)** day 1, **B)** day 3, and **C)** day 7 under direct contact conditions. (●/■) vs Control: ● $p \leq 0.05$; ■ $p \leq 0.01$.

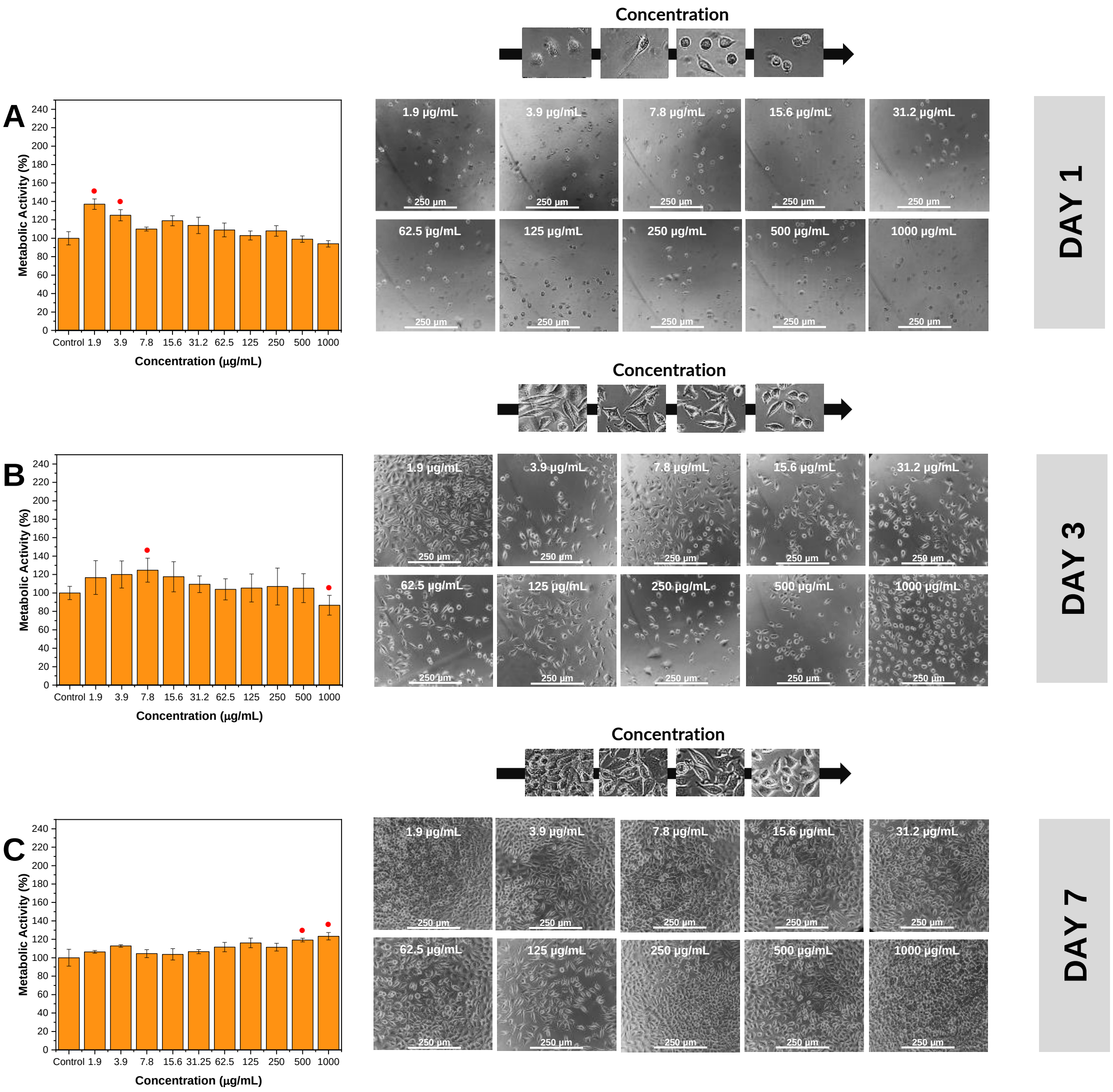

Figure S11. Cell metabolic activity via the MTT assay and optical microscopy in L929 cells exposed $CaWO_4$ at **A)** day 1, **B)** day 3, and **C)** day 7 under indirect contact conditions. (●/■) vs Control: ● $p \leq 0.05$; ■ $p \leq 0.01$.

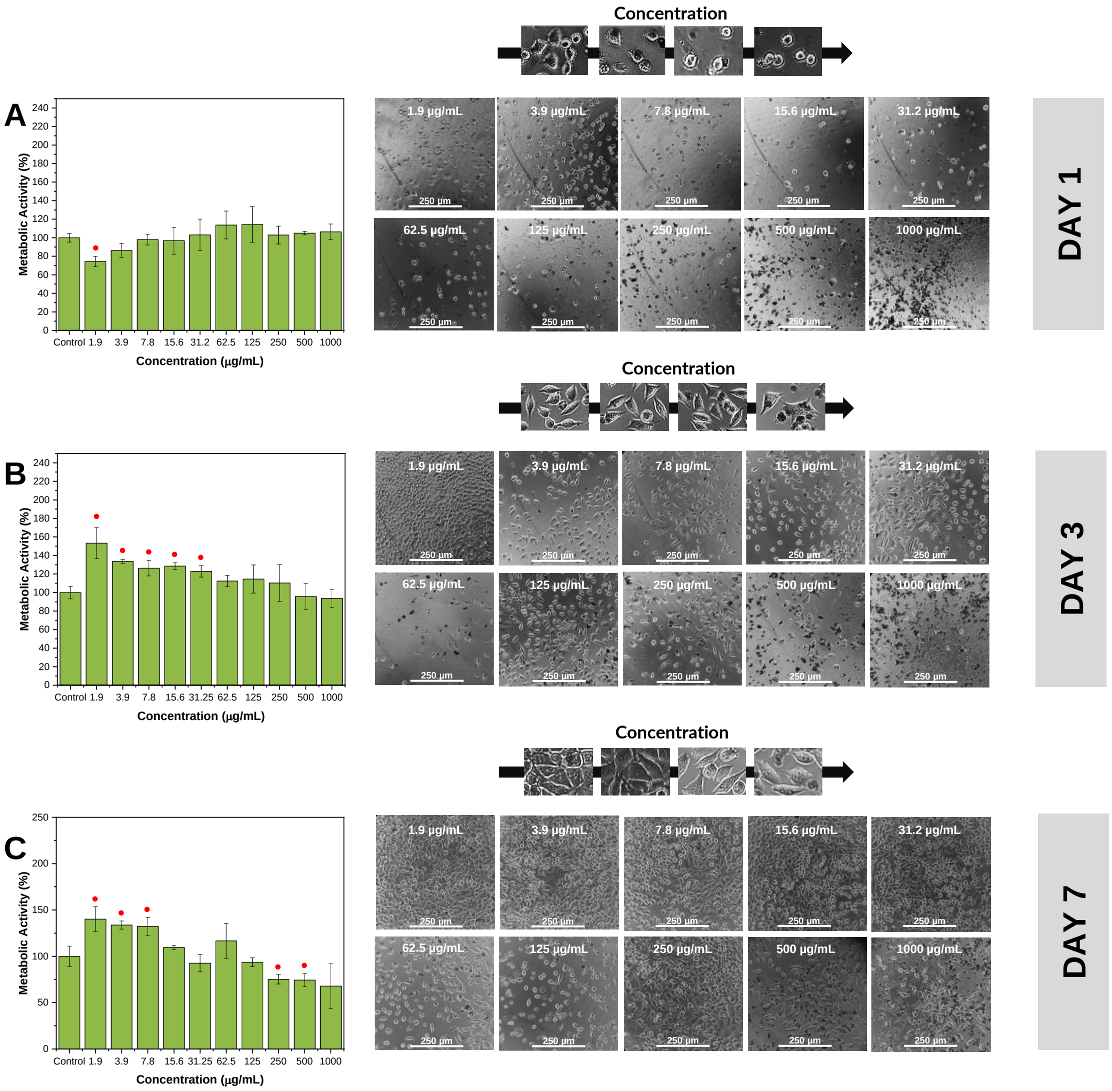

Figure S12. Cell metabolic activity assessed via the MTT assay and optical microscopy in L929 cells exposed $SrMoO_4$ at **A)** day 1, **B)** day 3, and **C)** day 7 under direct contact conditions. (●/■) vs Control: ● $p \leq 0.05$; ■ $p \leq 0.01$.

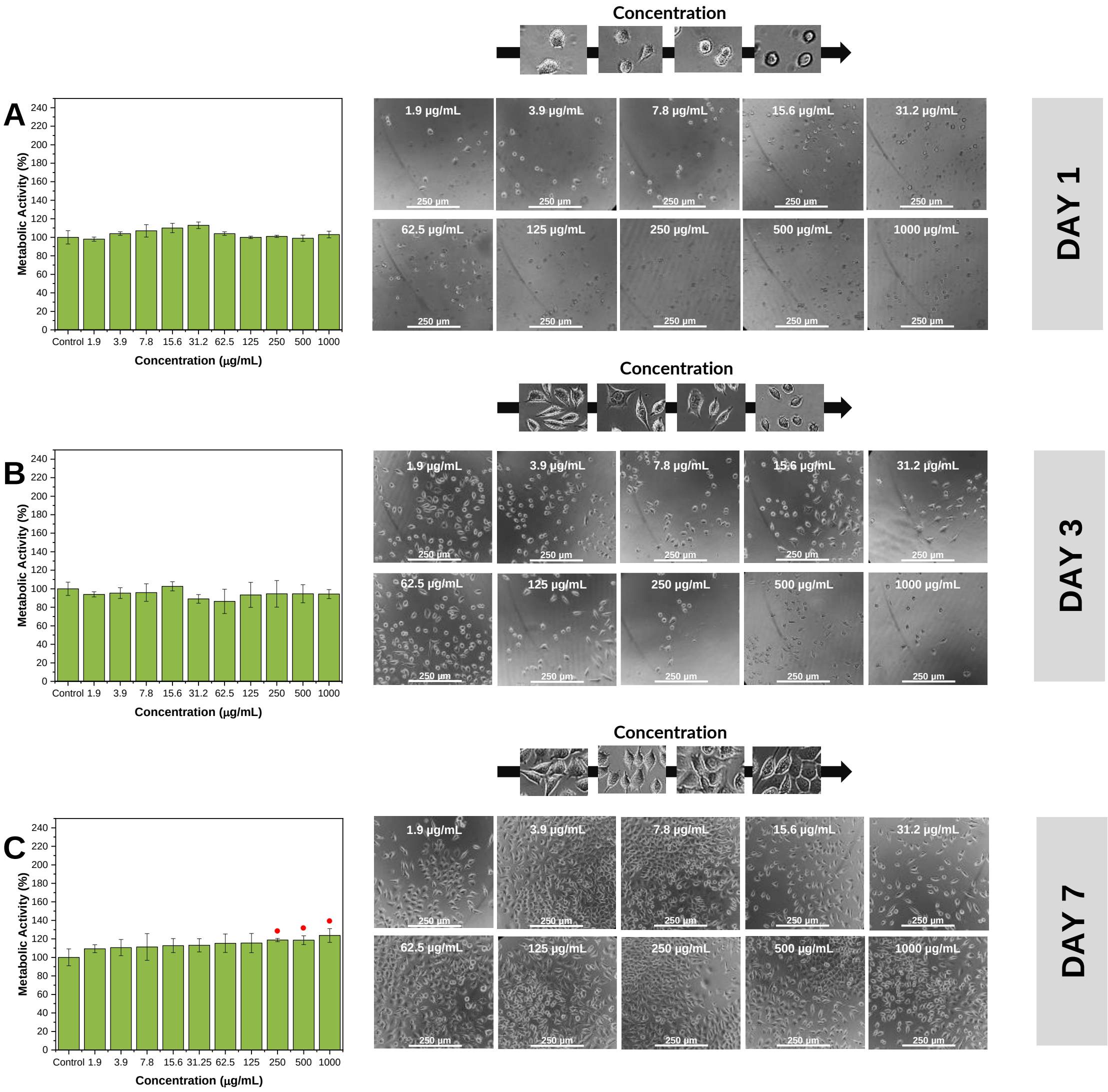

Figure S13. Cell metabolic activity via the MTT assay and optical microscopy in L929 cells exposed $SrMoO_4$ at **A)** day 1, **B)** day 3, and **C)** day 7 under indirect contact conditions. (●/■) vs Control: ● $p \leq 0.05$; ■ $p \leq 0.01$.

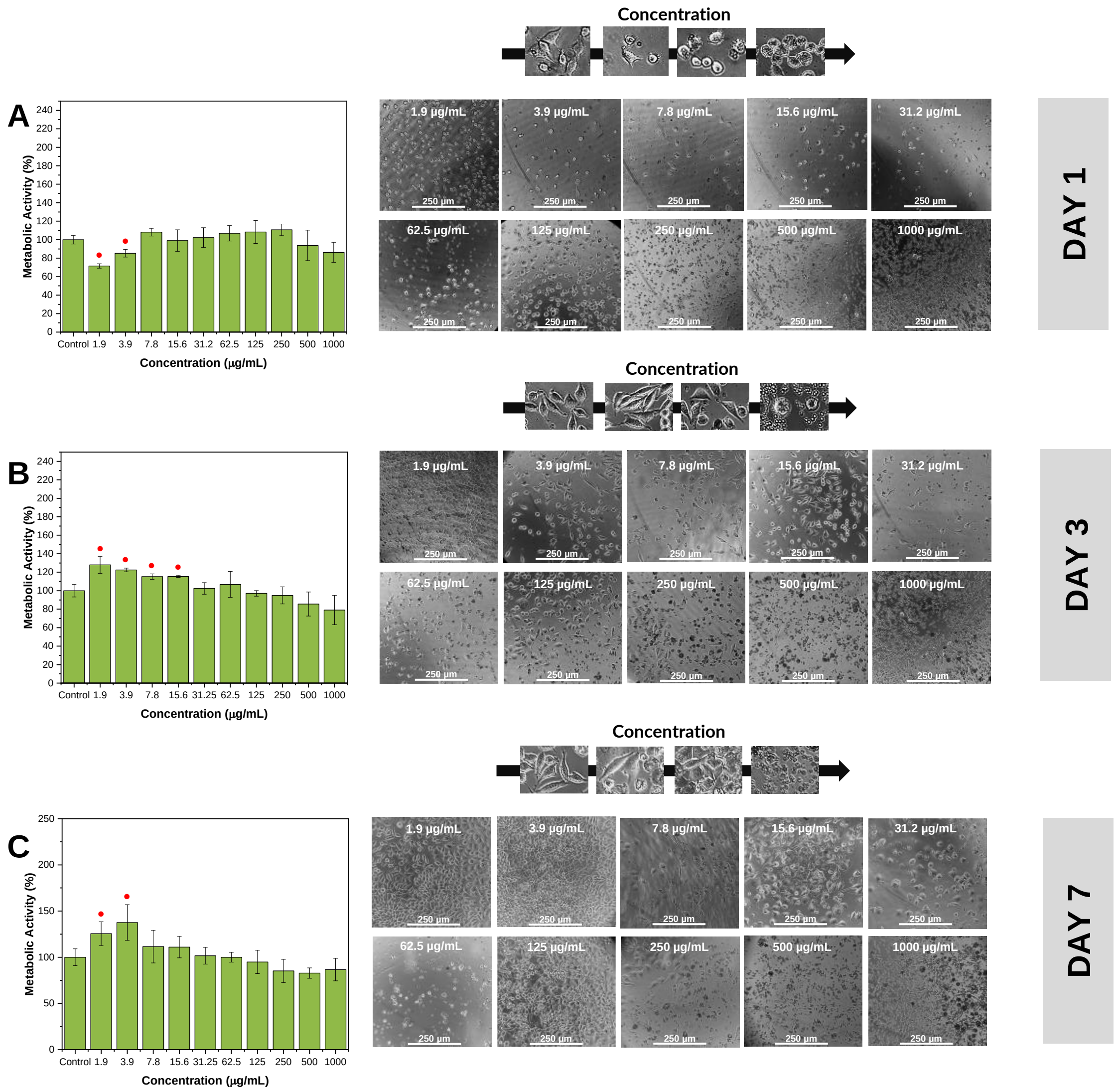

Figure S14. Cell metabolic activity via the MTT assay and optical microscopy in L929 cells exposed $SrWO_4$ at **A)** day 1, **B)** day 3, and **C)** day 7 under direct contact conditions. (●/■) vs Control: ● $p \leq 0.05$; ■ $p \leq 0.01$.

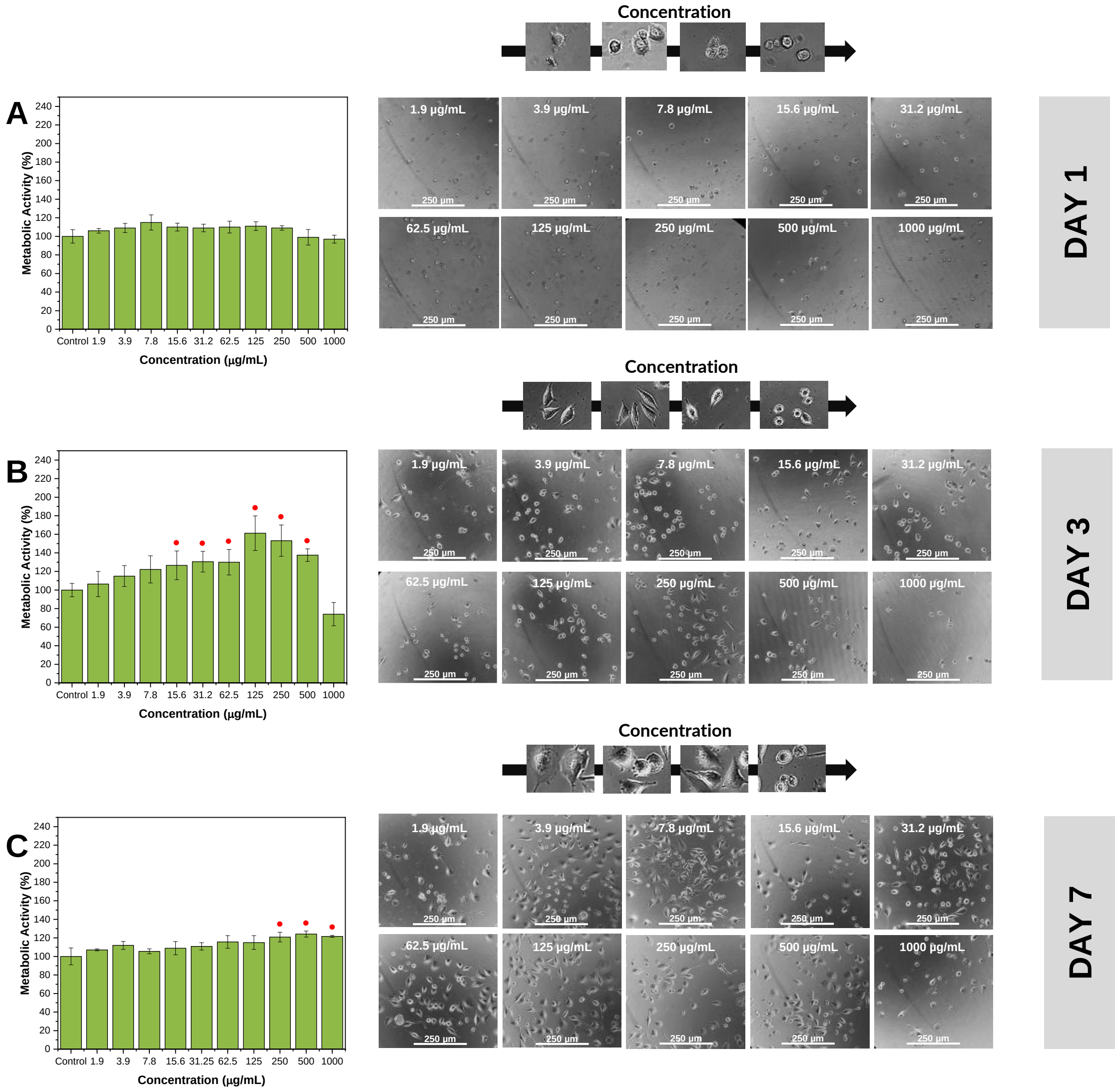

Figure S15. Cell metabolic activity via the MTT assay and optical microscopy in L929 cells exposed $SrWO_4$ at **A)** day 1, **B)** day 3, and **C)** day 7 under indirect contact conditions. (●/■) vs Control: ● $p \leq 0.05$; ■ $p \leq 0.01$.

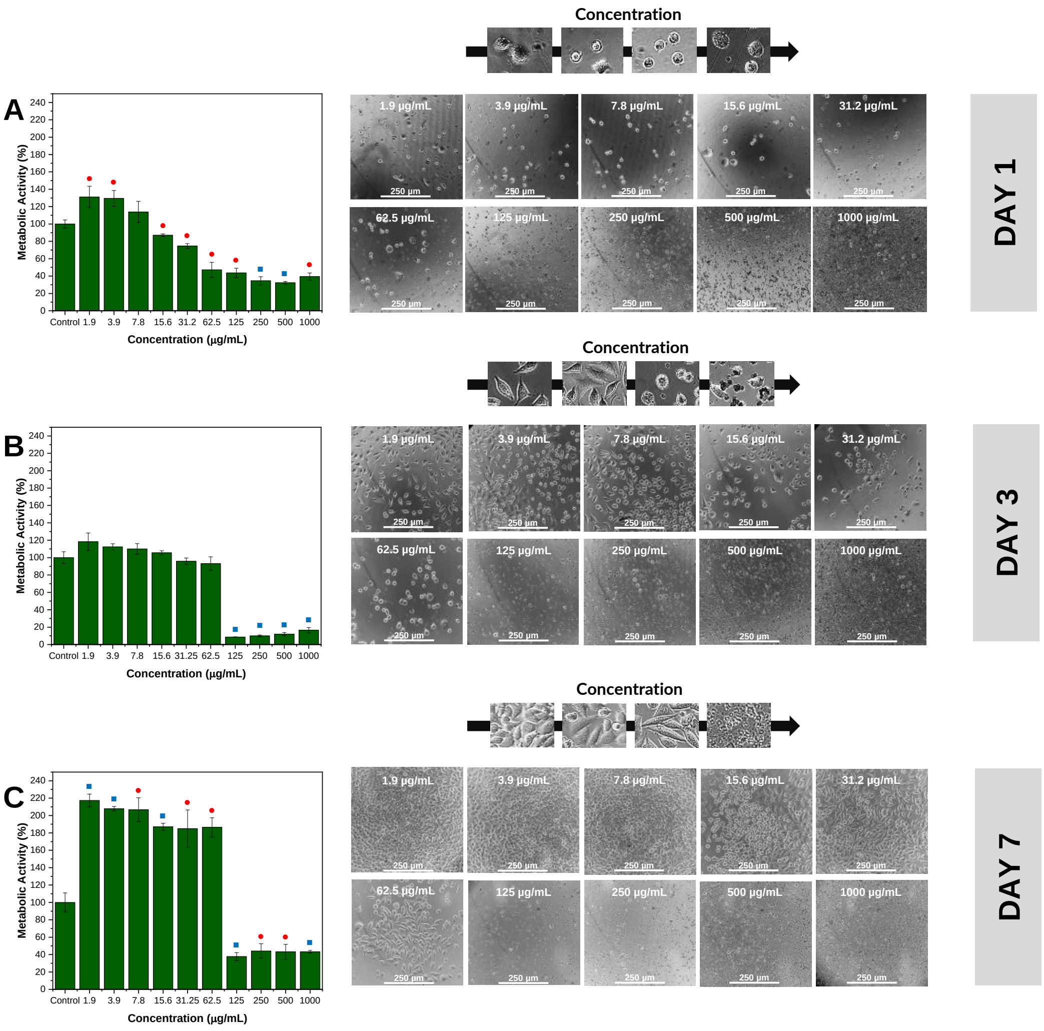

Figure S16. Cell metabolic activity via the MTT assay and optical microscopy in L929 cells exposed β-$ZnMoO_4$ at **A)** day 1, **B)** day 3, and **C)** day 7 under direct contact conditions. (●/■) vs Control: ● $p \leq 0.05$; ■ $p \leq 0.01$.

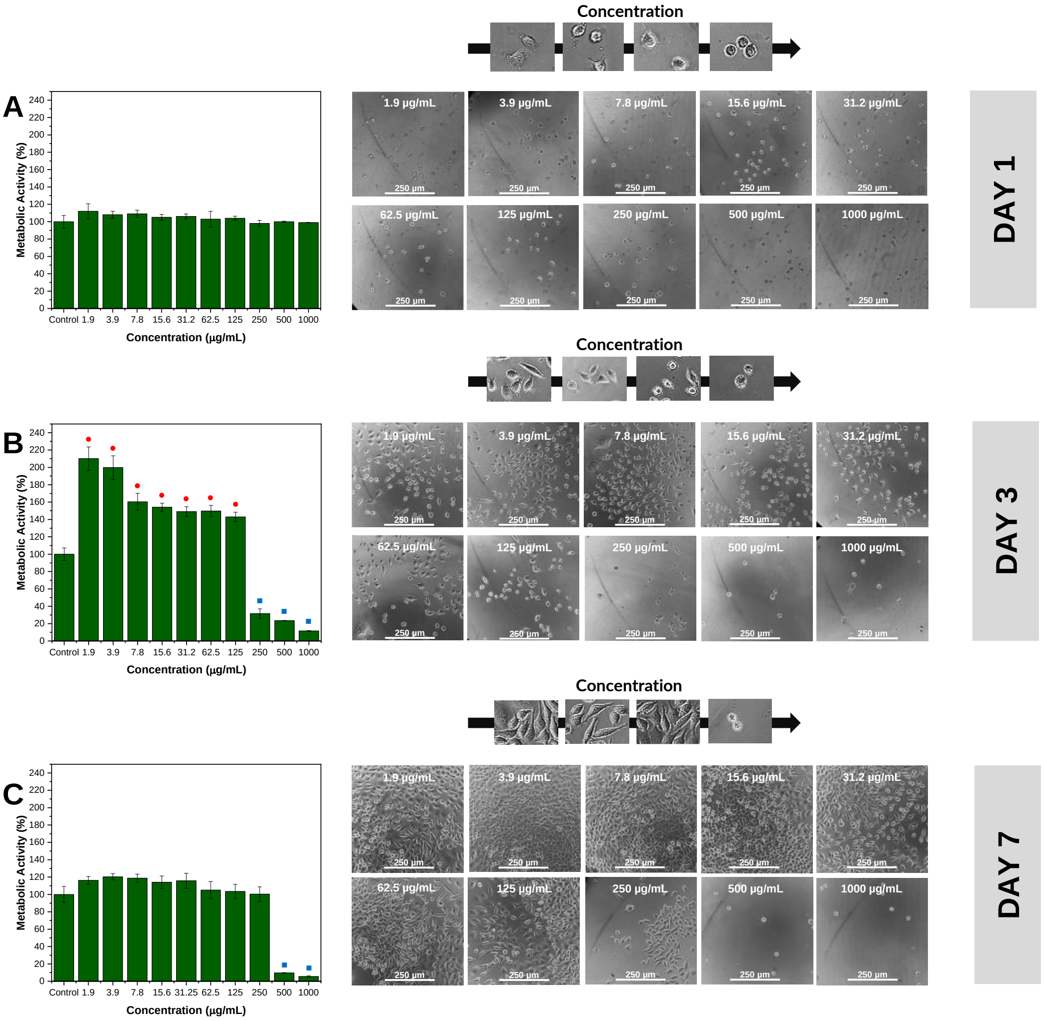

Figure S17. Cell metabolic activity via the MTT assay and optical microscopy in L929 cells exposed β-$ZnMoO_4$ at **A)** day 1, **B)** day 3, and **C)** day 7 under indirect contact conditions. (●/■) vs Control: ● $p \leq 0.05$; ■ $p \leq 0.01$.

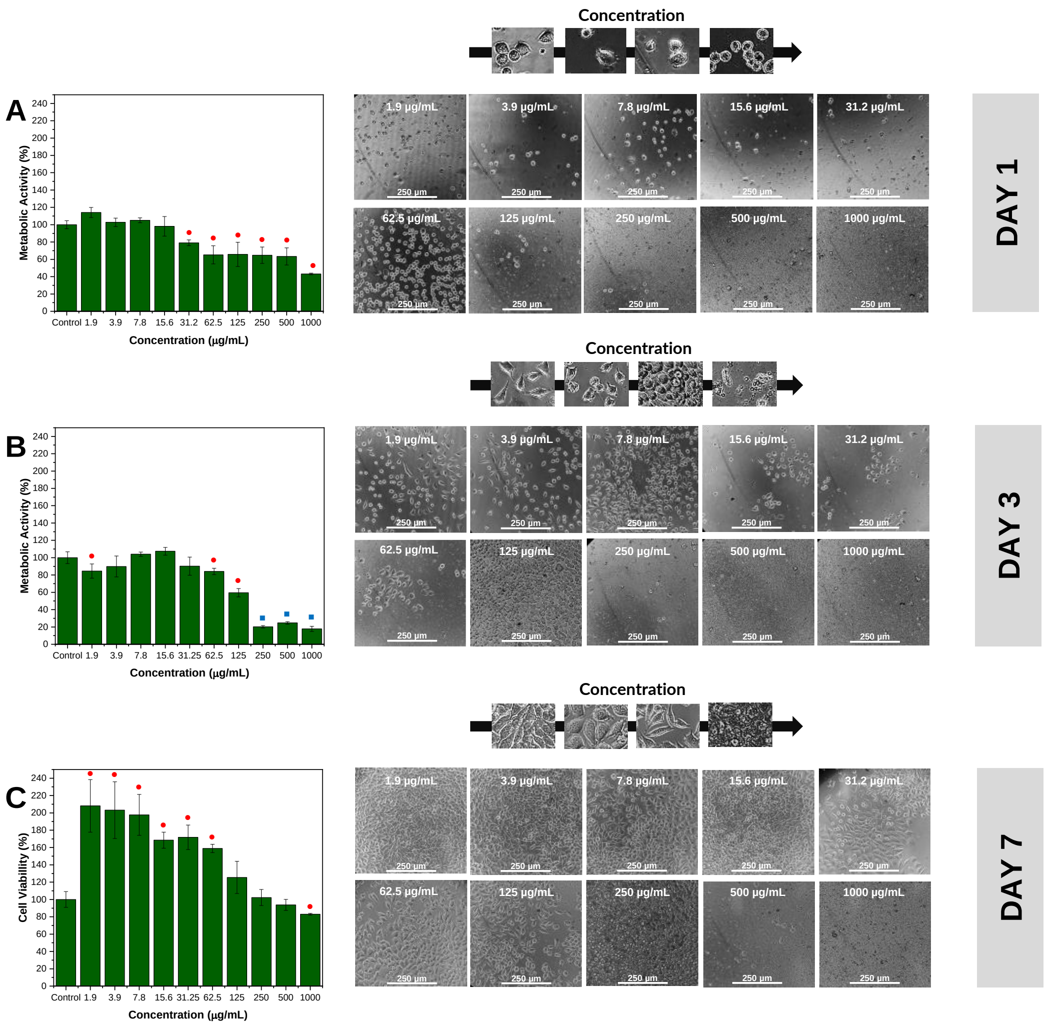

Figure S18. Cell metabolic activity via the MTT assay and optical microscopy in L929 cells exposed $ZnWO_4$ at **A)** day 1, **B)** day 3, and **C)** day 7 under direct contact conditions. (●/■) vs Control: ● $p \leq 0.05$; ■ $p \leq 0.01$.

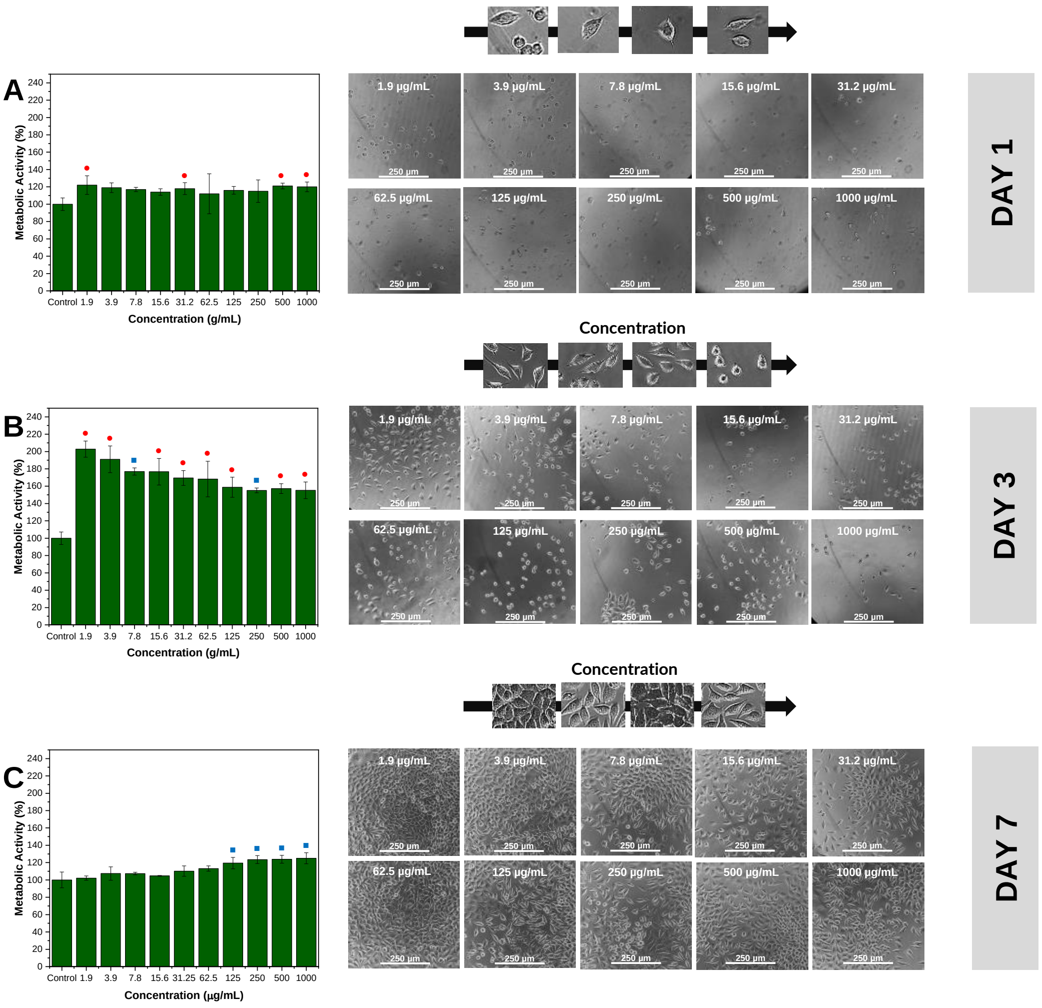

Figure S19 Cell metabolic activity via the MTT assay and optical microscopy in L929 cells exposed $ZnWO_4$ at **A)** day 1, **B)** day 3, and **C)** day 7 under indirect contact conditions. (●/■) vs Control: ● $p \leq 0.05$; ■ $p \leq 0.01$.

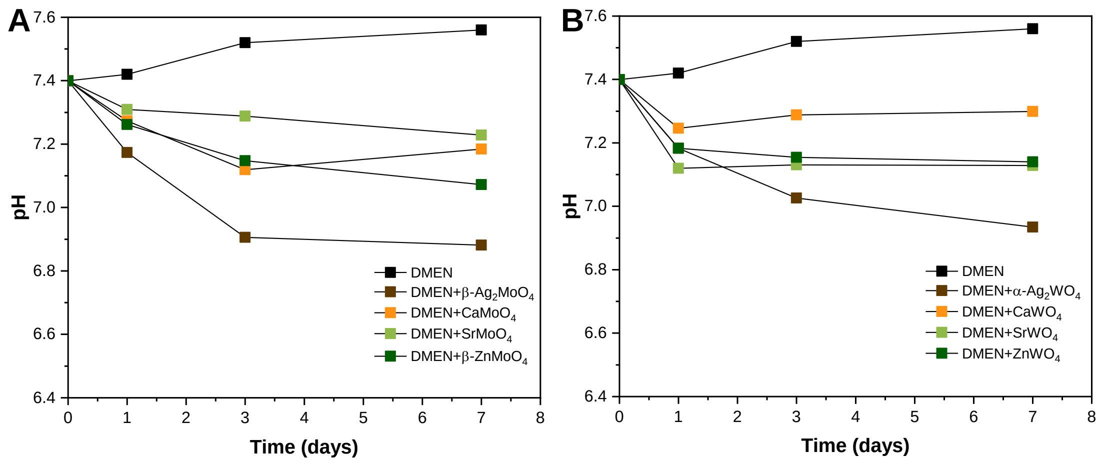

Figure S20. pH time evolution of DMEM cell medium with **A)** transition metal molybdites and **B)** transition metal tungstates.

Table S1. Ionic concentrations (µM) released into the medium after 24 hours of exposure for the different materials, measured by ICP analysis at the three lowest tested concentrations.

**Concentration (µg/mL)**	**β-Ag_2MoO_4**		**$CaMoO_4$**		**$SrMoO_4$**		**β-$ZnMoO_4$**	
	**Ag (µM)**	**Mo (µM)**	**Ca (µM)**	**Mo (µM)**	**Sr (µM)**	**Mo (µM)**	**Zn (µM)**	**Mo (µM)**
**1.9**	1.32	1.93	0.53	0.41	0.32	0.18	0.38	0.37
**3.9**	2.15	4.34	1.30	0.90	0.45	0.45	0.56	0.65
**7.8**	3.69	7.90	2.31	1.31	1.49	0.73	0.82	0.86
**Concentration (µg/mL)**	**α-Ag_2WO_4**		**$CaWO_4$**		**$SrWO_4$**		**$ZnWO_4$**	
	**Ag (µM)**	**W (µM)**	**Ca (µM)**	**W (µM)**	**Sr (µM)**	**W (µM)**	**Zn (µM)**	**W (µM)**
**1.9**	2.45	1.79	2.46	0.80	0.70	0.21	0.48	0.19
**3.9**	2.95	4.09	3.56	1.50	0.94	0.30	0.65	0.42
**7.8**	5.04	5.84	5.02	2.16	1.42	0.39	0.94	0.53

Table S2. Percentage of total ionic leaching relative to the initial ion content for the different materials after 24 hours of exposure, measured by ICP analysis at the three lowest tested concentrations.

**Inicial Concentration (µg/mL)**	**β-Ag_2MoO_4**			**$CaMoO_4$**			**$SrMoO_4$**			**β-$ZnMoO_4$**		
	**Ag (% w/w)**	**Mo (% w/w)**	**Total (% w/w)**	**Ca (% w/w)**	**Mo (% w/w)**	**Total (% w/w)**	**Sr (% w/w)**	**Mo (% w/w)**	**Total (% w/w)**	**Zn (% w/w)**	**Mo (% w/w)**	**Total (% w/w)**
**1.9**	7.49	9.77	17.26	1.12	2.09	3.21	0.47	0.89	1.36	1.31	1.87	3.18
**3.9**	5.95	10.67	16.63	1.34	2.20	3.54	0.33	1.10	1.42	0.94	1.61	2.55
**7.8**	5.11	9.72	14.83	1.19	1.61	2.79	0.54	0.89	1.43	0.68	1.05	1.74
**Concentration (µg/mL)**	**α-Ag_2WO_4**			**$CaWO_4$**			**$SrWO_4$**			**$ZnWO_4$**		
	**Ag (% w/w)**	**W (% w/w)**	**Total (% w/w)**	**Ca (% w/w)**	**W (% w/w)**	**Total (% w/w)**	**Sr (% w/w)**	**W (% w/w)**	**Total (% w/w)**	**Zn (% w/w)**	**W (% w/w)**	**Total (% w/w)**
**1.9**	13.92	17.31	31.23	5.19	7.73	12.92	1.04	2.05	3.09	1.64	1.87	3.52
**3.9**	8.15	19.30	27.45	3.66	7.05	10.71	0.68	1.41	2.08	1.09	1.98	3.07
**7.8**	6.97	13.76	20.73	2.58	5.09	7.67	0.51	0.93	1.44	0.79	1.25	2.04
